# Vicariant speciation resulting from biogeographic barriers in the Australian tropics: The case of the red‐cheeked dunnart (*Sminthopsis virginiae*)

**DOI:** 10.1002/ece3.70215

**Published:** 2024-08-27

**Authors:** Linette S. Umbrello, Hayley Newton, Andrew M. Baker, Kenny J. Travouillon, Michael Westerman

**Affiliations:** ^1^ School of Biology and Environmental Science Queensland University of Technology Brisbane Queensland Australia; ^2^ Collections and Research Western Australian Museum Welshpool Western Australia Australia; ^3^ School of Environmental and Conservation Sciences Murdoch University Murdoch Western Australia Australia; ^4^ Biodiversity and Geosciences Program Queensland Museum South Brisbane Queensland Australia; ^5^ Department of Ecology and Genetics La Trobe University Bundoora Victoria Australia

**Keywords:** biodiversity, dasyuridae, mammal, marsupial, phylogeography, sminthopsinae, sminthopsini

## Abstract

Global biodiversity loss continues unabated, and in Australia, the rate of recent mammal extinctions is among the worst in the world. Meanwhile, the diversity among and within many endemic mammal species remains undescribed. This information is crucial to delineate species boundaries and thus inform decision‐making for conservation. *Sminthopsis virginiae* (the red‐cheeked dunnart) is a small, dasyurid marsupial found in four disjunct populations around the northern coast of Australia and New Guinea. There are three currently recognized subspecies, each occupying a distinct geographic location. *Sminthopsis v. virginiae* occurs in Queensland, *S. v. rufigenis* is distributed across New Guinea and the Aru Islands, and *S. v. nitela* has populations in the Top End of the Northern Territory and the Kimberley region of Western Australia. Previous molecular work has suggested the current subspecies definitions are not aligned with DNA sequence data, though the sampling was limited. We undertook a comprehensive genetic and morphological review of *S. virginiae* to clarify relationships within the species. This included mitochondrial (CR, 12S, and cytb) and nuclear (omega‐globin, IRBP, and bfib7) loci, and morphometric analysis of skulls and whole wet‐preserved specimens held in museums. Maximum Likelihood and Bayesian phylogenetic analyses resolved samples into two distinct clades, demarcated by the Gulf of Carpentaria in Australia's north. *Sminthopsis. v. nitela* was consistently separated from *S. v. virginiae* and *S. v. rufigenis*, based on the overall body and skull size and craniodental features, while *S. v. virginiae* and *S. v. rufigenis* were more difficult to distinguish from each other. Thus, we redescribed *S. virginiae*, recognizing two species, *S. nitela* (raised from subspecies) and *S. virginiae* (now comprising the subspecies *S. v. virginiae* and *S. v. rufigenis*). This study highlights the importance of recognizing cryptic mammal fauna to help address the gap in our knowledge about diagnosing diversity during a time of conservation crisis.

## Graphical abstract

We investigated the diversity of red‐cheeked dunnarts (*Sminthopsis virginiae*) of northern Australia and New Guinea using molecular and morphological data. We found that the subspecies *S. v. nitela* was genetically and morphologically more distinct than the eastern subspecies, *S. v. rufigenis* and *S. v. virginiae*, and thus raised it to full species status.

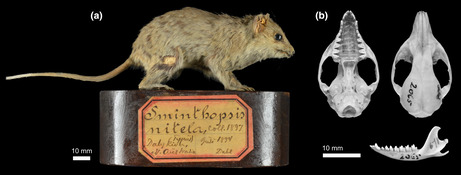

## 1 INTRODUCTION

Rates of global biodiversity loss continue to accelerate in the 21st century as human impacts on natural ecosystems worsen. In Australia, the anthropogenic impacts on biodiversity have been profound. Changes brought about by European colonization have wrought devastation on various flora and fauna, in particular a suite of endemic, unique, and isolated mammals (Woinarski et al., 2015). Currently, 149 mammal taxa and populations are listed as threatened under the Australian Environment Protection and Biodiversity Conservation Act 1999 (Department of Climate Change, Energy, Environment and Water, 2024), 40 of which are considered to be extinct (Burbidge, 2023). More recently, declining mammal populations have been documented in regions previously thought to be relatively undisturbed, such as northern Australia (Cremona et al., 2022; Woinarski et al., 2011), which is of particular concern as species in this region are typically understudied. Documenting the diversity of Australian mammals is now critical as the cumulative effects of multiple threats are realized, such as eroding natural habitats coupled with widespread bushfires and predation by introduced species such as feral domestic cats (*Felis catus*) and European red foxes (*Vulpes vulpes*).

Among Australia's mammal fauna are the carnivorous dasyurid marsupials, comprising about 61 species (AMTC, 2023), many of which are restricted to peripheral or sensitive habitats and predicted to continue to decline during this century (Krajewski et al., 2024). The subfamily Sminthopsinae comprises almost half of the extant dasyurid species, and the majority of these belong to the genus *Sminthopsis*, one of only four currently recognized genera. Three of these: *Antechinomys* (two species), *Ningaui* (three species), and *Sminthopsis* (18 species) compose the tribe Sminthopsini; the fourth (*Planigale*) constitutes the tribe Planigalini (Krajewski et al., 2024). Although both morphological and molecular studies have consistently shown the monophyly of both tribes, several phylogenetic issues are in need of resolution, including the monophyly of *Sminthopsis* as currently recognized (Krajewski et al., 2012). Blacket et al. (2001) summarized the history of phylogenetic studies of Sminthopsins until that time and demonstrated that the precise interrelationships, and even the composition, of the three currently recognized genera were unclear since *Antechinomys* and *Ningaui* rendered *Sminthopsis* paraphyletic in all molecular studies. This, and all subsequent studies (Blacket et al., 2006; García‐Navas & Westerman, 2018; Kealy & Beck, 2017; Krajewski et al., 2012; Westerman et al., 2016) have consistently demonstrated that the long‐tailed dunnart, *S. longicaudata*, is sister to *Antechinomys laniger*, while the remaining species of *Sminthopsis* belong to one or other of two distinct species groups: “*macroura*” and “*murina*.” Following analysis of DNA sequences from both mitochondrial and nuclear genes, Westerman et al. (2023) suggested that the long‐tailed dunnart (previously *S. longicaudata*) be recognized as a second species of *Antechinomys* (now *A. longicaudatus*).

Krajewski et al. (2012) extended the existing molecular dataset for Sminthopsins and incorporated some new characters based on penis morphology, demonstrating that the “*macroura*” species group was distinguishable not only by their DNA sequences but also by all members sharing a unique phallic morphology (Form 1, Woolley et al., 2007). However, status and interrelationships of some of the species currently recognized in this group (*Sminthopsis crassicaudata, S. bindi, S. douglasi, S. macroura*, and *S. virginiae*) remain unclear. Although some species such as *S. bindi* and *S. douglasi* have relatively limited distributions, others are widespread across northern and central Australia and show variability not only in their external morphology but also in DNA sequence and allozyme (isozyme) profiles across the species' range (see Archer, 1981; Blacket et al., 2001; Umbrello et al., 2020). Some of these distinctive features have been used to designate currently recognized subspecies: two for *S. crassicaudata* (*S. c. crassicaudata* and *S. c. centralis*); at least three for *S. macroura* (*S. m. macroura, S. m. froggatti, S. m. stalkeri*) and three for *S. virginiae* (*S. v. virginiae, S. v. nitela*, and *S. v. rufigenis*). Some of the currently recognized subspecies were formerly recognized as distinct taxa before Archer (1981) synonymized them in his major review of the genus *Sminthopsis*. Thus, *S. virginiae* specimens collected from Cape York Peninsula, Queensland, were originally described as *Phascogale virginiae* de Tarragon, 1847; specimens from the Daly River region of the Northern Territory were named *Sminthopsis nitela* Collett, 1897; and specimens from the Aru Islands and the Trans Fly region of southern New Guinea were described as *S. rufigenis* Thomas, 1922.

The genetic variation within *Sminthopsis virginiae* has been investigated in only one study (Blacket et al., 2001). Two genetic clades were resolved, Western Australia (WA) + Northern Territory (NT) and Queensland (Qld) + Papua New Guinea (PNG), using two mitochondrial loci (12S and Control Region [CR]) but only a small number of individuals. The authors concluded the divergence between the two clades was similar to that observed between other species of *Sminthopsis*, and as such, *S. v. nitela* was genetically distinct enough to warrant raising to species.

In Archer's (1981) review of the genus *Sminthopsis*, he stated that “other than by its larger size” *S. v. virginiae* could not easily be distinguished from *S. v. nitela*. However, it differed from *S. v. rufigenis* in having females with eight nipples, instead of six, and for being generally larger and possessing a larger alisphenoid tympanic wing on the skull. *Sminthopsis v. nitela* differed from both other subspecies by smaller body size and the lack of an anterior cingulum on the upper molar teeth. However, Archer's (1981) investigations were muddied by the sample of “*nitela*” from NT including *S. butleri* (at the time, the latter was only known from WA), the inclusion of an undescribed *S. bindi* and museum specimen NTMU4340 (which was later identified as *Pseudantechinus bilarni*, by P. G. Horner in 1996), as well as specimen AM M4403, a *S. macroura*.

In this study, we investigate the interrelationships between the three currently recognized subspecies of *Sminthopsis virginiae*, greatly expanding the DNA sequence dataset and conducting a detailed morphological analysis of samples across the known geographic range of the species.

## 2 MATERIALS AND METHODS

### 2.1 Study species

**FIGURE 1:**
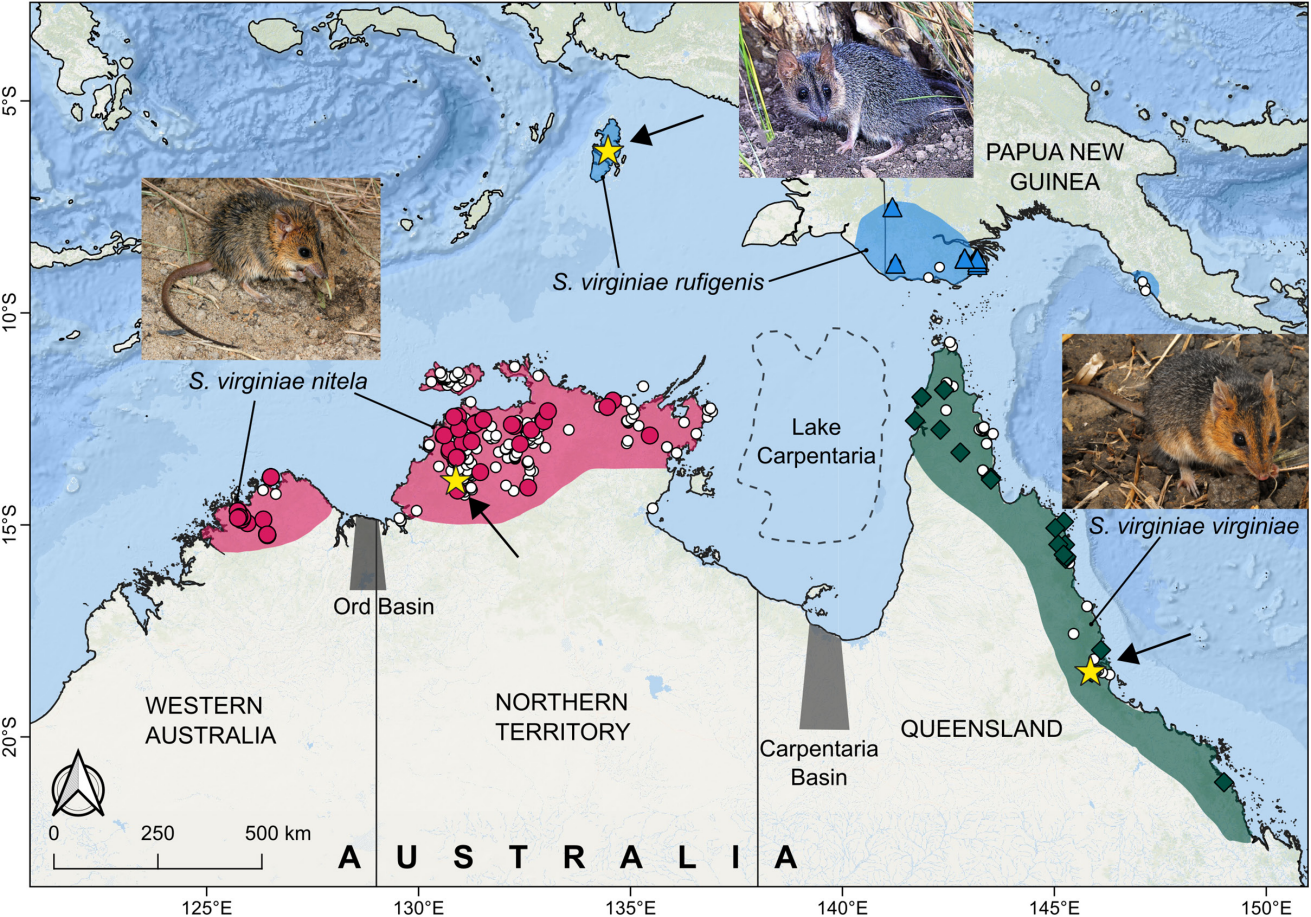
Map showing the distribution of *Sminthopsis virginiae* records (white circles) and samples used in this study (filled shapes) in northern Australia and southern Papua New Guinea (PNG), along with the localities from which type specimens were collected (yellow stars). The approximate species distribution is shown as shaded areas, colored by each subspecies. The approximate location of biogeographic barriers is shown with gray rectangles and labeled, as is the approximate location of the historical Lake Carpentaria (demarcated by dotted lines). Photos: *S. v. nitela*, Anders Zimny; *S. v. virginiae*, Eric Vanderduys; *S. v. rufigenis*, Pat Woolley.

*Sminthopsis virginiae* is a small (18–60 g), predominantly insectivorous marsupial that occurs in disjunct populations across northern Australia, southern New Guinea, and the Aru Islands (Figure 1). They prefer tropical forest and savannah woodlands with heavy soils in swampy and riparian areas with dense vegetation (Bradley et al., 1987; Braithwaite & Lonsdale, 1987; Tate & Archbold, 1941). The species' common name, the red‐cheeked dunnart, alludes to the rufous fur patches on the sides of the head and neck, which distinguish it from all other *Sminthopsis* species, but the extent of this facial coloration and overall dorsal fur color is variable. Each of the three currently recognized subspecies occupies distinct, non‐overlapping geographic regions (Krajewski et al., 2024; Woolley, 2023) (Figure 1). Differences in the nipple number of females, six in *S. v. rufigenis* and eight in *S. v. virginiae* and *S. v. nitela*, the presence or absence of an anterior cingulum on the upper molar teeth, and overall body size, are thought to be among the few diagnostic characters that can be used to distinguish between the three subspecies (Archer, 1981).

### 2.2 Specimens sampled

**FIGURE A1:**
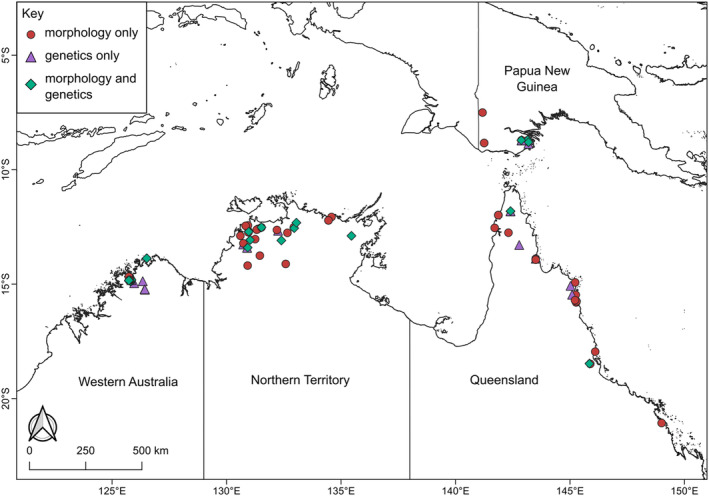
Map of sample localities for *Sminthopsis virginiae* with type of material examined coded as genetic data only (triangle), morphological data only (circle), or as genetic and morphological data available from the same specimen (diamond).

In addition to two exemplars of each of the currently recognized species included in the “*macroura*” species group of *Sminthopsis*, we included 58 specimens of each recognized sub‐species of *S. virginiae* to clarify genetic relationships within this species. Specimens sampled are shown in Figure 1, and sample details and genes sequenced are listed in Appendix A; Table A1. Figure A1 shows on a map which samples were used for molecular and morphological studies.

### 2.3 Molecular methods

Total genomic DNA was extracted from museum preserved liver, muscle, and ear tissue samples cryo‐preserved at −80°C or stored in 70%–100% ethanol using Qiagen DNeasy® tissue and blood kits according to manufacturer's instructions, and genetic loci were amplified as described in Umbrello et al. (2017) at the Western Australian Museum. Primer details and PCR cycling are available as supplementary methods (see File S2). Bi‐directional Sanger DNA sequencing was carried out at the Australian Genome Research Facility (Perth, Western Australia). Alignment of sequence reads was performed in Geneious Prime (Kearse et al., 2012) as described in Umbrello et al. (2017) and Westerman et al. (2023). We amplified 181 new DNA sequences from three mitochondrial (Control Region [CR] *n* = 29, *l* = 558 bp, 12S rRNA [12S] *n* = 50, *l* = 972 bp, Cytochrome *b* [cytb] *n* = 43, *l* = 1146 bp) and three nuclear (omega globin [ω‐globin] *n* = 41, *l* = 822 bp, β fibrinogen intron 7 [bfib7] *n* = 15, *l* = 1454, interphotoreceptor binding protein [IRBP] *n* = 4, *l* = 1068 bp) loci. The new cytb, ω‐globin, and IRBP gene sequences were checked for authenticity by looking for premature stop codons, frameshift mutations, indels, etc., as well as for indications of pseudogene amplification. GenBank accession numbers for the novel sequences generated in this study include PP851402–PP851404, PP853397–PP853441, PP854425, and PP900178 for 12S, PP858677–PP858705 for CR, PP887888–PP887930 for cytb, PP858706–PP858743 and PP898284–PP898286 for ω‐globin, PP887931–PP887945 for bfib7 and PP898280–PP898283 for IRBP (see Table A1). “Outgroups” for the molecular studies included the representatives of each species of the “*macroura*” group.

### 2.4 Molecular analyses

The DNA sequence matrix was analyzed using Maximum Likelihood (implemented in RAxML 7.2.8, Stamatakis, 2006) and Bayesian methods (implemented in MrBayes 3.2, Ronquist et al., 2012). Data were separated into six gene partitions, three nuclear (IRBP, ω‐globin, and bfib7) and three mitochondrial (cytb, 12SrRNA, CR) regions, each with its own model of sequence evolution as determined by jModeltest (Posada, 2008). In addition, we further partitioned protein‐coding genes into their three codon positions (first, second, and third), and the two rRNA genes into stems and loops, to determine whether this affected resolution in the phylogenetic tree and if the topology of this tree differed from others. Branch lengths were not partitioned in our analyses. The general time‐reversible model with gamma‐distributed rates and a fraction of invariable sites (GTR + G + I) was used for all partitions in RAxML. For Bayesian analysis, best‐fit models (see Table A2) were chosen using the Akaike Information Criterion as implemented in jModeltest 2.1.7 (Posada, 2008). Node support was estimated by 1000 bootstrap pseudoreplications for RAxML. Bayesian analyses utilized random starting trees and two simultaneous runs of four Markov chains (one cold and three heated, using default heating values) applied for 5 × 10^6^ generations, with sampling every 1000th generation. The first 1.25 × 10^6^ generations were discarded from each run as burn‐in. The remaining trees were used to construct a maximum clade credibility tree; posterior probabilities (PP) >0.95 were deemed as strong support and PP = 0.90–0.95 as moderate support (see Kolaczkowski & Thornton, 2006). User‐trees were tested using PAUP* (Swofford, 2003).

Average sequence divergence levels among samples of *Sminthopsis virginiae* and the outgroup taxa were determined using PAUP*. Kimura two‐parameter (K2P) corrected distance values were calculated for all positions in cytb and transversions only for 12S (Kimura, 1980). Using these results, divergence estimates were calculated for cytb based on the estimated divergence from Krajewski et al. (2000) of 2.1% per million years for *Sminthopsis* which assumed a 25 million year date for the divergence of thylacinids and dasyurids, and for 12S following the method outlined in Springer et al. (1997) where divergence time (My) = (% divergence – 0.0584)/0.0854 where % divergence is the K2P value (for transversions only) in the 12S rRNA gene.

In this study, we have adopted the Unified Species Concept of De Queiroz (2007) which proposes reciprocal monophyly as providing evidence for species boundaries, and we tested for reciprocal monophyly using the Species Delimitation plugin in Geneious Prime (Masters et al., 2011). This tool assesses the strength of the phylogenetic evidence of species boundaries by applying Rosenberg's *P*_*AB*_ statistic (Rosenberg, 2007), which was developed for calculating the probability of reciprocal monophyly under the null model of random coalescence. Rosenberg's *P*_*AB*_ statistic is the probability that *A* putative species represented by *a* taxa will be monophyletic relative to a sister clade containing *b* taxa under the null model of random coalescence.

To investigate broad‐scale phylogeographic patterns in the sequence data, statistical parsimony TCS haplotype networks (Clement et al., 2000) were constructed using the software program PopART (Population Analysis with Reticulate Trees; Leigh & Bryant, 2015) where each subspecies was well represented by the sequence data. To obtain haplotypes for the nuclear ω‐globin sequence data where individuals were heterozygotes we used the program PHASE v. 2.1.1 (Stephens et al., 2001) and created input files using the webtool SeqPHASE (Flot, 2010). We ran PHASE using default settings and only included phased haplotypes with confidence scores greater than 90%. PopART excludes gaps and unknown bases (N, ? and –) from the analysis, so these regions were removed from the alignments before building networks. Networks were built for each loci separately due to different individuals having coverage for different loci, with samples colored based on population location: WA, NT, Qld, or PNG.

### 2.5 Morphology

To assess the morphological variation among *Sminthopsis virginiae* subspecies, 93 vouchered specimens (which included 33 skulls, 16 skins, and 68 wet preserved specimens) were examined from the following institutions: Australian National Wildlife Collection, CSIRO (ANWC); Queensland Museum (QM); Western Australian Museum (WAM), the Museum and Art Gallery of the Northern Territory (MAGNT) and the Natural History Museum at the University of Oslo (NHMO) (see Figure A1 for the geographic distribution of material examined). Additionally, photographs of type specimens were examined from the Natural History Museum, UK and the Natural History Museum at the University of Oslo, Norway. Skulls and dentition were examined to identify and corroborate diagnostic features as described in Archer (1981). Similarly, pelt coloration was examined on dry skins and wet preserved specimens to identify any differences between the subspecies. Female spirit specimens were examined for pouch development, nipple number, and the presence of pouch young.

**FIGURE 2:**
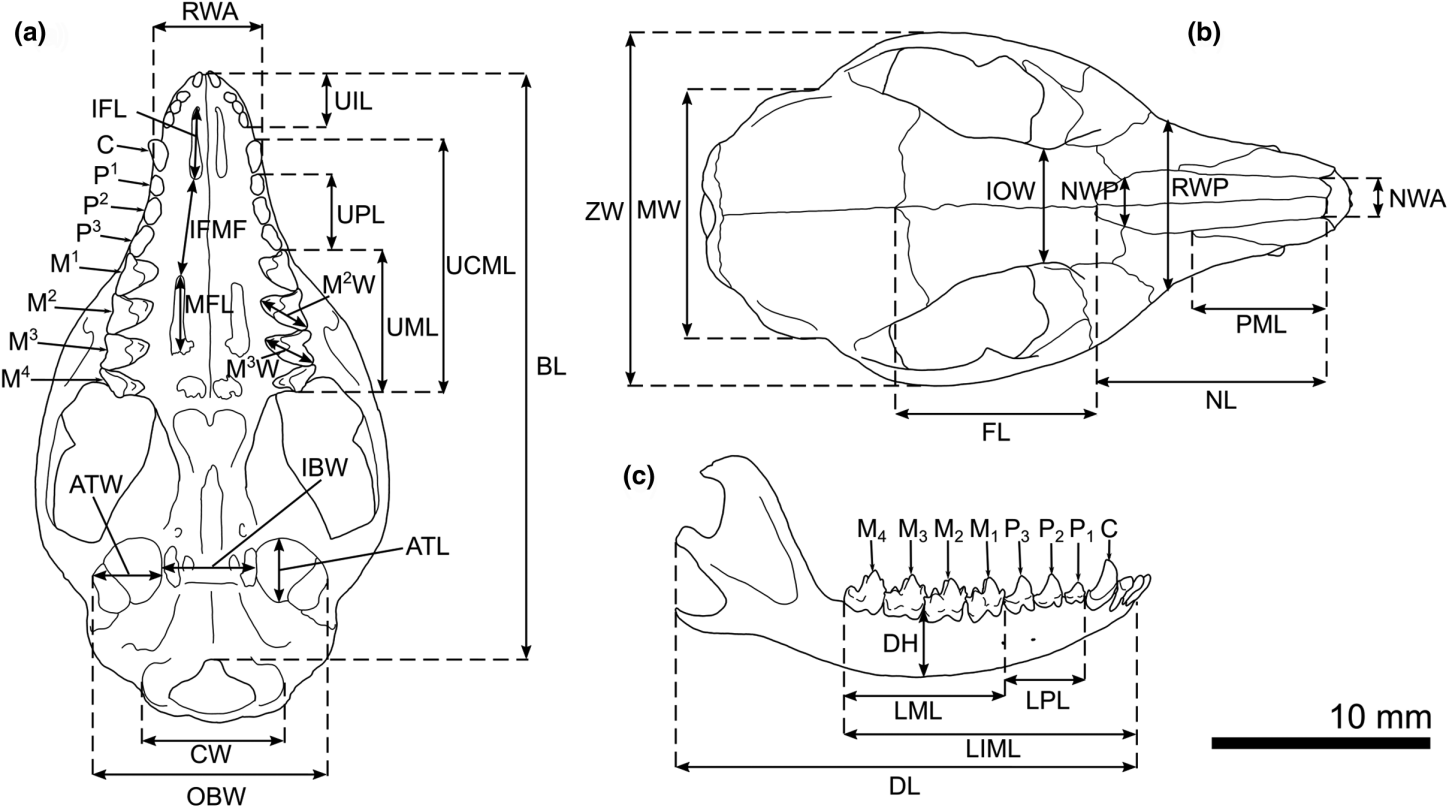
Illustration of the *Sminthopsis virginiae*, based on ANWC M29776, cranium (a,b) and dentary (c) with measurements and teeth labeled.

Tooth numbering followed Luckett (1993), and anatomical terminology of teeth and cranial features followed Beck et al. (2022). Skull measurements are illustrated in Figure 2 and were taken from Archer (1981), Baker et al. (2015), and Umbrello (2018). The measurements used were as follows: ATL, alisphenoid tympanic process length; ATW, alisphenoid tympanic process width; BL, basicranial skull length, excluding incisors; CW, combined width of occipital condyles; DH, dentary height measured between M_2_ and M_3_; DL, dentary length, excluding incisors; FL, length of frontal midline suture; IBW, minimum width between auditory bullae; IFL, length of incisive foramen; IFMF, distance between the incisive foramen to the maxillopalatine fenestra; IOW, minimum width of interorbital constriction; LIML, maximum length of lower tooth row from I_1_ to M_4_; LML, maximum length of lower molar row, M_1–4_; LPL, crown length of lower premolar row P_1–3_; M^2^W, maximum width of upper M^2^, measured at crown; M^3^W, maximum width of upper M^3^, measured at crown; MFL, length of maxillopalatine fenestra; MW, maximum width across braincase; NL, maximum length of nasals; NWA, anterior width of nasals; NWP, width of nasals at the nasal/maxilla/frontal junction; OBW, maximum outer width of auditory bullae; PML, length of premaxilla, measured as length of sutural contact between nasal bone and premaxilla; RWA, rostral width, from the outer edge of each upper canine; RWP, rostral width, across the medial surface of each infraorbital foramina; UCML, maximum length of upper tooth row from canine to M^4^; UIL, maximum length of upper incisor series, I^1–4^; UML, maximum length of upper molar row, M^1–4^; UPL, crown length of upper premolar row P^1–3^; ZW, maximum zygomatic width. The following external measurements were taken from preserved (wet) specimens: head‐vent length, taken from the tip of the nose to the vent; tail‐vent length, from the vent to the tip of the tail; ear length, measured across the longest part of the ear from the tip to the notch; pes length, from the heel to longest toe tip (minus claw); and head length, from the tip of the nose to the back of the cranium. All skull and external measurements are given in mm.

Skulls were categorized as adults if M4 was fully erupted and the deciduous premolar, dP3, had been lost and replaced by a fully emergent P3 (Archer, 1981). Individuals that did not fall into the adult class were excluded from the morphometric analyses. Where wet‐preserved bodies were present, sexual maturity was confirmed by the development of the external reproductive structures (i.e., presence and size of testes [males] or pouch and nipple development [females]), and juvenile or subadult individuals were not included in analyses. In total, we measured 19 *S. v. nitela*, seven *S. v. rufigenis*, and seven *S. v. virginiae* extracted skulls, which represented all the available material in Australian institutions. Due to the small number of individuals available of *S. v. rufigenis* and *S. v. virginiae*, sexes were grouped for all analyses; while males are generally larger than females for many dasyurid species, the differences are slight for the smaller members of the genus (see body sizes reported in Baker & Gynther, 2023).

All measurements were recorded by hand with digital calipers to 0.01 mm (Table A3 and File S1), and statistical analyses were carried out in the computing program R (R Core Team, 2013) using a custom script that incorporated elements of the “MorphoTools2” package (Šlenker et al., 2022). Univariate statistics and analysis of variance (ANOVA) were conducted on the three subspecies for each measurement variable where data were normally distributed and homogeneity of variances tested with Bartlett's test (Bartlett, 1937). One variable (ATL) did not have equal variances, so a nonparametric Kruskal–Wallis test (Kruskal & Wallis, 1952) was run instead. Pairwise comparisons for significant ANOVA results were obtained with Tukey's post hoc test (Tukey, 1949) with correction for multiple comparisons. This allowed us to identify which measurements were most informative for identifying diagnostic features between the subspecies.

For multivariate analyses, variables with greater than 20% missing data were removed; this cut‐off was chosen to reduce the amount of missing data while maximizing the number of measurement variables retained in the dataset. Any individuals with more than 15% missing data after this initial step were subsequently removed (these were ANWC M29861, 28% missing, MAGNT U3769, 20% missing, and WAM M4056, 16% missing). We accounted for the multicollinearity of variables via Pearson correlation coefficients, and JL and ZW were removed due to being highly correlated (>90%–95%) with BL. Hierarchical clustering using Euclidean distance and the UPGMA algorithm was conducted on the remaining dataset to see if the morphological distance was clustered according to subspecies.

The remaining missing data (only three variables M^2^W, M^3^W, and DH for one individual, QMJ15890) were imputed using predictive mean matching in the “MICE” package (Buuren and van Buuren & Groothuis‐Oudshoorn, 2011) adopting the default settings, except that we increased the number of iterations from five to 50. To visualize the imputed craniodental measurement data, we conducted a Principal Component Analysis (PCA) using the pca.cor function in “MorphoTools2”. In morphological datasets, a high proportion of the variance explained by PC1 is usually due to size (Berner, 2011; Jolicoeur, 1963), and accounting for the effect of size has been shown to help distinguish between morphologically cryptic dasyurid species as it allows for comparison on skull shape (Umbrello et al., 2023; Viacava et al., 2023). To account for size effects (specifically caused by isometric scaling) between species, we used log‐shape ratios by calculating the geometric mean of all variables, per individual, as a proxy for skull size, dividing each variable by the geometric mean, then log‐transforming this ratio (Mosimann, 1970; Onley et al., 2022; Viacava et al., 2023). The PCA was then re‐run on the size‐corrected data.

Differences in overall skull size between the subspecies were tested using ANOVA on the geometric means. This was followed by a Tukey's post hoc test to check the pairwise differences between groups with p‐values adjusted for multiple group testing. To test how well individuals could be placed into the correct subspecies grouping, we used nonparametric k‐nearest neighbor classificatory Discriminant Analysis due to the small sample sizes of *Sminthopsis v. rufigenis* and *S. v. virginiae*, nonequal covariances and deviation from multivariate normality. We ran this analysis on four different sets of data to assess the effect of size correction on correctly classifying specimens to subspecies and whether the genetic clades were better at diagnosing specimens than the current subspecies hypotheses.

## 3 RESULTS

### 3.1 Molecular analyses

**FIGURE 3:**
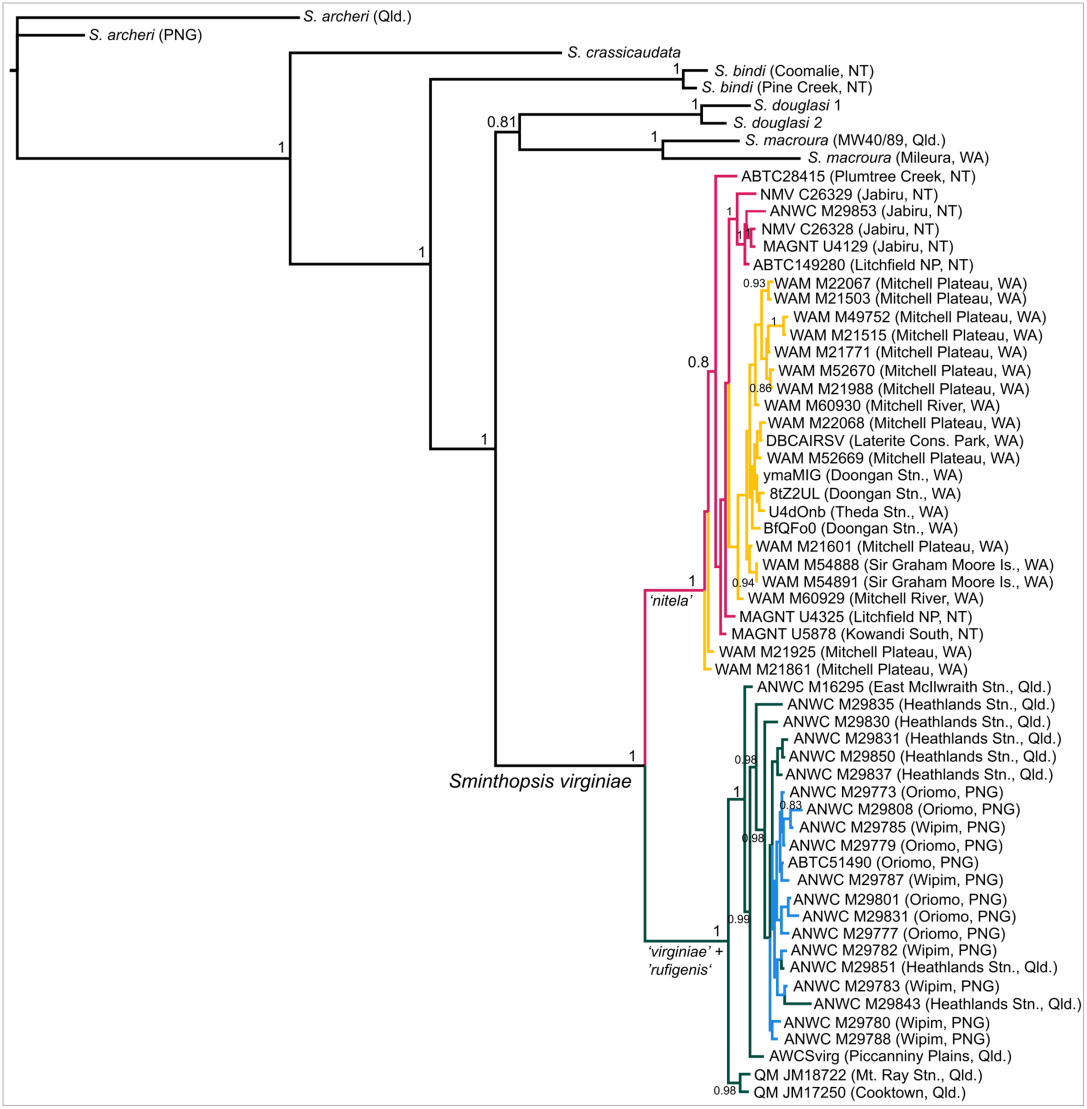
Bayesian tree of concatenated nuclear and mitochondrial gene loci partitioned by gene locus and codon position (RY). Sample subspecies identification: Northern Territory (NT) *Sminthopsis virginiae nitela* populations (pink); Western Australia (WA) *S. v. nitela* populations (yellow); Queensland (Qld) populations of *S. v. virginiae* (green); Papua New Guinea (PNG) populations of *S. v. rufigenis* (blue). Numbers at nodes are Bayesian Posterior Probabilities (BPP) >0.81.

Our Maximum Likelihood and Bayesian analyses of the partitioned concatenated datasets provided a well‐resolved, monophyletic group comprising all specimens of *Sminthopsis virginiae* (Figure 3), which consisted of two well‐resolved subgroups: one comprising all individuals from the Northern Territory (NT) and the Kimberley region of Western Australia (WA); the other comprising all individuals from Cape York Peninsula, Queensland (Qld) together with those from the Trans‐Fly region of southern Papua New Guinea (PNG). Our tree thus resolved the currently recognized subspecies *S. v. nitela* as sister to a lineage comprising specimens of *S. v. virginiae* and *S. v. rufigenis*, though neither of the latter was themselves monophyletic. Both clades were well supported with Bootstrap support >94% for RAxML or Bayesian Posterior Probability of 1.00.

**FIGURE A2:**
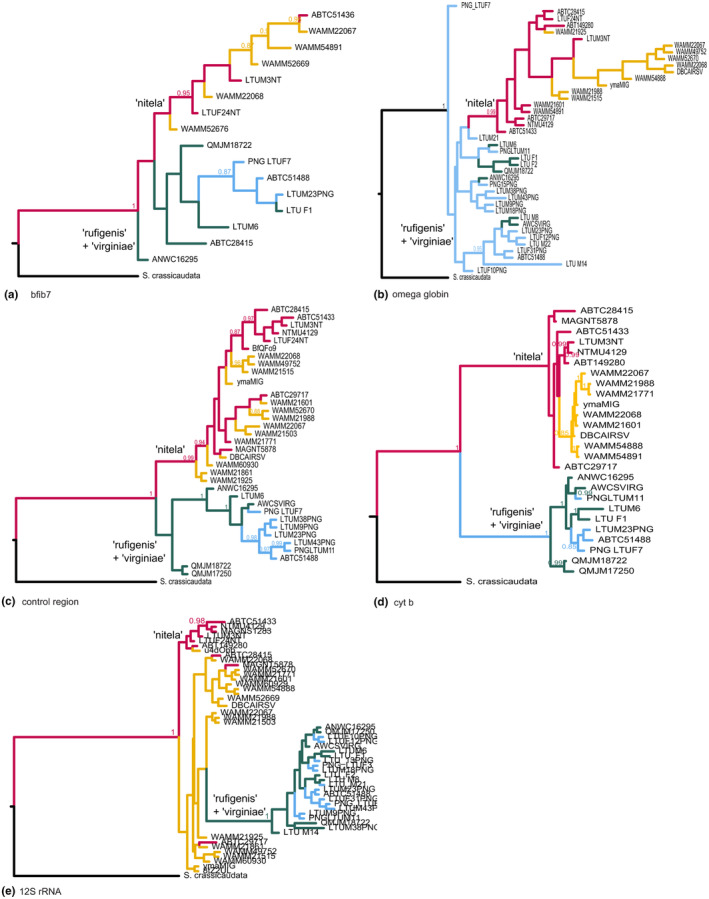
Single gene trees, (a) β fibrinogen intron 7, (b) omega globin, (c) Control Region, (d) Cytochrome (b,e) 12 s rRNA. Samples from the Kimberley, WA yellow; NT, red; Qld, green and PNG, blue.

The topology of this tree did not differ from the single gene trees (Figure A2), as all showed a separation of *Sminthopsis virginiae* samples into two distinct clades: one comprising specimens from the Northern Territory and Western Australia (“*nitela*”) and one comprising those from Queensland and Papua New Guinea (“*virginiae*” + “*rufigenis*”). Within this latter clade, there was no obvious distinction between exemplars of the two currently recognized subspecies, and a user‐tree specifying such a separation was a significantly worse fit to the data (Table 1).

**TABLE 1:** Probabilities of specified user‐tree tests in PAUP*.

Tree	‐Lnl	Diff‐Lnl	KH	Weighted KH	SH	Weighted SH	AU
(“*nitela*,” (“*virginiae*” + “*rufigenis*”))	18041.00	Best					
((“nitela” NT, “nitela” WA), (“*virginiae*” + “*rufigenis*”))	18063.20	22.200	0.118	0.118	0.408	0.142	0.008
(“*nitela*,” (“*virginiae”*, “*rufigenis*”))	18219.37	178.376	*p* < .0005***	*p* < .0005***	*p* < .0006***	*p* < .0006	~0*
((“*nitela*” NT, “*nitela*” WA), (“*virginiae*,” “*rufigenis*”))	18241.63	200.633	*p* < .0002***	*p* < .0002***	*p* < .0002***	*p* < .0002	~0*

Abbreviations: AU, Shimodaira Approximately Unbiased test; KH, Kishino–Hasegawa Test using RELL bootstrap (2‐tailed test); Lnl, log likelihood; SH, Shimodaira–Hasegawa Test using RELL bootstrap (1‐tailed test).

We evaluated the fit of our data to alternative phylogenetic relationships using the SH (Shimodaira & Hasegawa, 1999) and KH (Kishino & Hasegawa, 1989) tests implemented in PAUP*4.0b10 (Swofford, 2003). These included: (1) that the currently recognized subspecies “*virginiae*” (Qld) and “*rufigenis*” (PNG) were distinct entities (i.e., reciprocally monophyletic), and (2) that within “*nitela*,” Northern Territory individuals were genetically distinct from Kimberley (WA) ones, as might be expected if the Ord Basin was effective in separating the two lineages over time. The results (Table 1) clearly show that the first of these maximum likelihood trees is a significantly worse fit to the data (*p* < .001*** for KH, wtd KH, SH, wtd SH, and AU tests) than the tree shown in Figure 3, suggesting that Queensland and Trans‐Fly Plains (PNG) populations of *S. virginiae* are genetically indistinguishable and are not monophyletic entities. The user‐tree positing genetic differences between NT and WA samples of “*nitela*” was not significantly different from the best tree (*p* > .05 for KH, wtd‐KH, SH, and wtd‐SH tests), suggesting that NT and WA “*nitela*” specimens are not clearly genetically divergent.

Thus, our mitochondrial and nuclear DNA sequence data suggest that *Sminthopsis virginiae* currently comprises two genetically distinct groups: the first of these comprises individuals currently recognized as *S. virginiae nitela*; the second comprises individuals currently recognized as belonging to *S. v. virginiae* and *S. v. rufigenis*, but which are genetically indistinguishable from one another based on the genetic markers examined.

### 3.2 Mitochondrial divergence

To further test the differences between currently recognized species and subspecies of *Sminthopsis virginiae*, we examined their differentiation using Kimura 2 Parameter (K2P) corrected distances derived from cytb gene sequences (Table 2). Corrected pairwise K2P differences between *S. virginiae* and the outgroup *Sminthopsis* species ranged from ~14%–20%. Within *S. virginiae*, K2P values for cytb were lower between *S. v. rufigenis* and *S. v. virginiae* (1.2%) compared to *S. v. nitela* (8%–8.2%), and values between the WA and NT populations of *S. v. nitela* (1.4%) were comparable to those between the eastern subspecies (1.2%). These correspond to average divergence times of ~3.9 million years (range = 2.88–5.11) for the Eastern and Western *virginiae* clades, and ~ 0.6 million years within each population, based on a 2.1% divergence per million years for dasyurids at cytb (Krajewski et al., 2000). For 12S, the K2P values were much smaller, being calculated based on transversions only, following the method described in Springer et al. (1997). The 12S K2P values reveal similar estimates, with divergences between the NT and WA, and Qld and PNG groups approximately 0.6 million years, and divergence between the Eastern and Western clades occurring 1.8–3.9 million years ago (where divergence time (My) = (% divergence – 0.0584)/0.0854).

**TABLE 2:** Average Kimura 2 Parameter corrected genetic distances (as percentages) between outgroup taxa and subspecies of *Sminthopsis virginiae*.

	*S. crassicaudata*	*S. macroura*	*S. bindi*	*S. douglasi*	*S. virginiae*	“nitela”	“rufigenis”	“virginiae”
*S. crassicaudata*	–	19.24	20.10	19.59	19.26			
*S. macroura*	1.818	–	16.38	14.33	14.63			
*S. bindi*	1.924	0.744	–	17.70	18.16			
*S. douglasi*	1.926	1.115	1.279	–	14.68			
*S. virginiae*	1.867	0.594	0.685	0.873	–			
“nitela”						–	8.09	8.23
							(6.05–10.74)	(6.05–10.0)
“rufigenis”						0.165	–	1.21
						(0.00–0.714)		(0.00–1.95)
“virginiae”						0.093	0.071	–
						(0.00–0.619)	(0.00–0.532)	

*Note*: Above diagonal cytb, below diagonal 12S. Ranges of values are shown in parentheses for comparisons within *S. virginiae* subspecies.

### 3.3 Species delimitation

The species delimitation analysis showed that *nitela* consistently formed a monophyletic clade, as did the combined *rufigenis* + *virginiae* samples, but not *rufigenis* nor *virginiae* on their own (Table 3). The ratio of intra‐distance versus inter‐distance showed low levels of genetic differences within the *nitela* sample relative to *rufigenis* + *virginiae*, but higher levels of within sample differences between *rufigenis* and *virginiae*, and for 12S these were equal to the differences between the two samples. Similarly, the probability of sorting samples of *nitela* into the correct clade was much higher (81%–95%) than sorting samples of *rufigenis* and *virginiae* (43%–79%). The *p*‐values for Rosenberg's *P*_*AB*_ were all significant for the *nitela* versus *rufigenis* + *virginiae* clades.

**TABLE 3:** Results from the species delimitation analysis run in Geneious on the concatenated tree and single gene trees with sufficient sample sizes: Cytb, 12S, and omega globin.

Tree	Species 1	Species 2	Mono	Intra/inter	Prob correct	Rosenberg's *P*_*AB*_
Concatenated	*nitela*	*rufigenis+virginiae*	Yes	0.19	0.93 (0.88, 0.98)	<0.001
	*rufigenis*	*virginiae*	No	0.55	0.79 (0.72, 0.86)	NA
Cytb	*nitela*	*rufigenis+virginiae*	Yes	0.13	0.95 (0.89, 1.00)	<0.001
	*rufigenis*	*virginiae*	No	0.65	0.75 (0.68, 0.81)	NA
12S	*nitela*	*rufigenis+virginiae*	Yes	0.28	0.90 (0.85, 0.96)	<0.001
	*rufigenis*	*virginiae*	No	1.00	0.43 (0.34, 0.51)	NA
ω‐globin	*nitela*	*rufigenis+virginiae*	Yes	0.60	0.81 (0.76, 0.87)	0.01
	*rufigenis*	*virginiae*	No	0.84	0.55 (0.47, 0.64)	NA

*Note*: For each pair of “species” or clades, whether they are monophyletic (Mono) is stated, the ratio of intra to inter genetic distance (Intra/Inter) between the species and the mean probability (Prob correct) with confidence intervals of placing an individual in the correct clade, and Rosenberg's *P*_*AB*_ value. Not calculated for groups that are not monophyletic.

### 3.4 Haplotype network analyses

**FIGURE 4:**
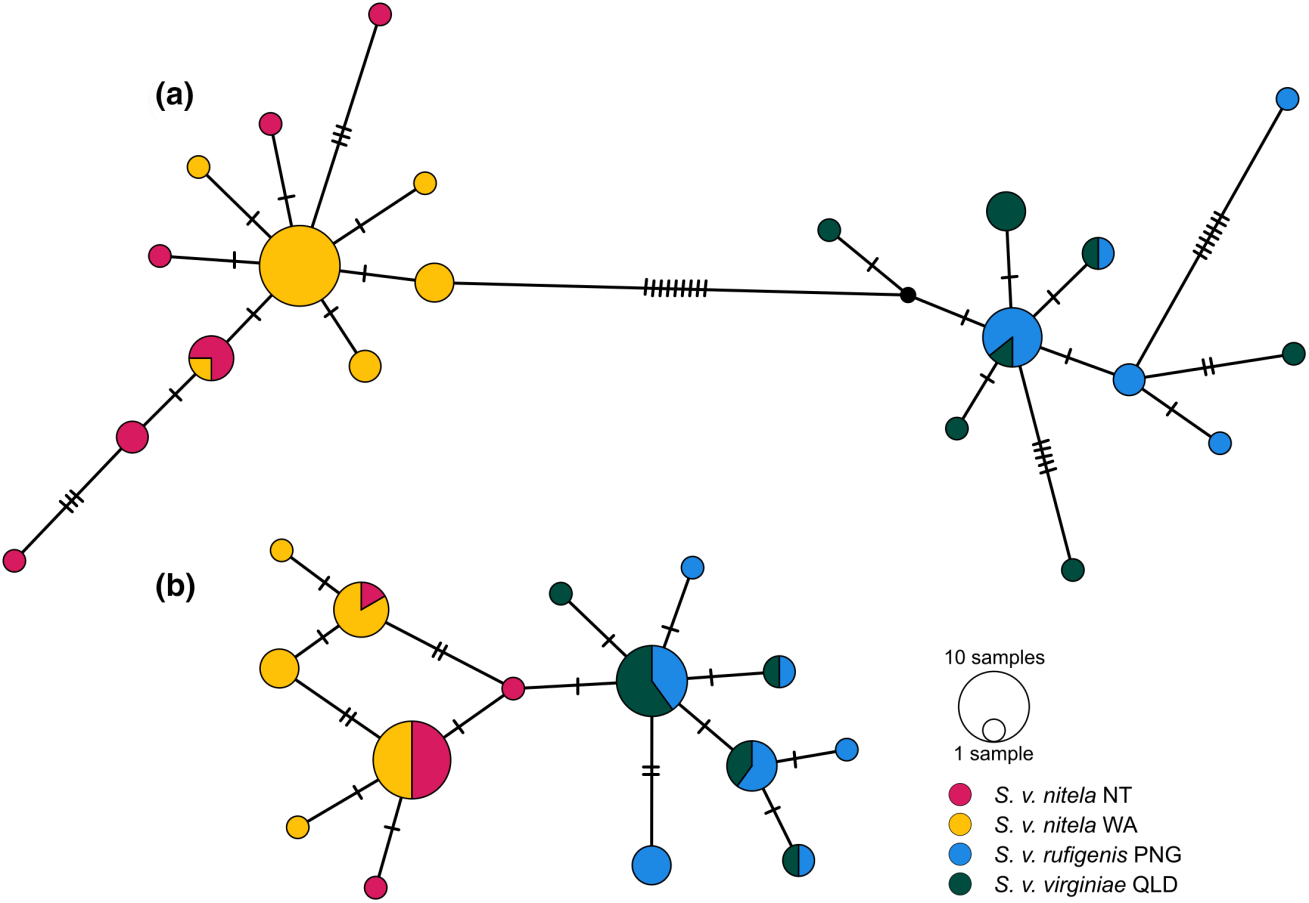
Parsimony (TCS) haplotype network of *Sminthopsis virginiae* (a) 12S sequences (*n* = 50, *l* = 567 bp) and (b) omega globin haplotypes (*n* = 50, *l* = 737 bp). Refer to the key within the figure for subspecies designation of the samples, tick marks represent nucleotide differences between unique haplotypes.

**FIGURE A3:**
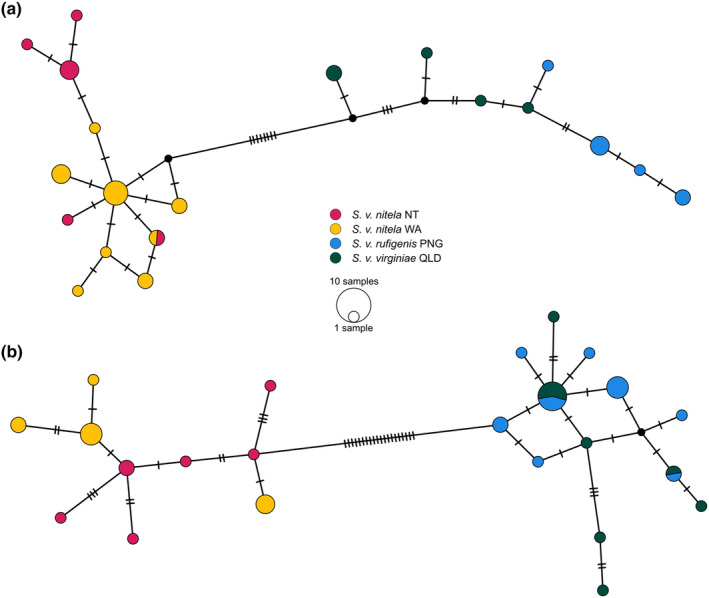
*Sminthopsis virginiae* mitochondrial haplotype networks for (a) control region (*n* = 35, *l* = 386 bp) and (b) cytochrome *b* (*n* = 41, *l* = 389 bp). Tick marks indicate nucleotide changes between samples haplotypes.

The length of the fragments used in the 12S network analysis was 567 bp, for CR it was 386 bp, for cytb, it was 389 bp, and for ω‐globin, it was 757 bp. All four networks resolved the samples into two groups consistent with the phylogenetic analyses (Figure 4; Figure A3). Some haplotypes were shared between NT and WA populations of *Sminthopsis v. nitela* in the 12S, CR, and ω‐globin data, and shared haplotypes were present between *S. v. rufigenis* and *S. v. virginiae* for 12S, cytb, and ω‐globin.

### 3.5 Cranial morphology

All variables were normally distributed for each subspecies, with the largest measurements recorded for the *Sminthopsis v. rufigenis* specimens (Table 4). One‐way ANOVAs showed significant differences between subspecies, except for the length of the nasals (NL and PML) and width of the auditory bullae (ATW and OBW) (see Table A4). Of these, 22 of the 27 measurements tested showed significant differences between *S. v. rufigenis* and *S. v. nitela*, 17 were significantly different between *S. v. virginiae* and *S. v. nitela* and only one measurement was significantly different between *S. v. virginiae* and *S. v. rufigenis* (FL, length of the frontal suture).

**TABLE 4:** Summary of cranial measurements (in mm) of *Sminthopsis virginiae* measured in this study.

Subspecies		BL	FL	NL	PML	NWA	NWP	RWP	IOW	ZW	UIL
*S. v. nitela*	*n*	18	19	19	19	19	19	19	19	19	19
	*x¯* ± *σ*	24.71 ± 1.30	8.77 ± 0.47	9.83 ± 0.91	5.77 ± 0.79	1.74 ± 0.28	2.49 ± 0.31	6.79 ± 0.44	5.48 ± 0.45	15.45 ± 0.86	2.52 ± 0.11
	range	22.99–27.97	7.90–9.63	8.23–11.59	3.91–6.99	1.23–2.38	2.01–3.24	5.84–7.70	4.74–6.41	13.95–17.15	2.33–2.76
*S. v. rufigenis*	*n*	7	7	7	7	7	7	7	7	7	7
	*x¯* ± *σ*	27.19 ± 1.68	9.84 ± 0.71	10.33 ± 0.54	5.92 ± 1.01	2.1 ± 0.15	2.85 ± 0.13	7.64 ± 0.41	6.11 ± 0.36	16.5 ± 1.11	2.81 ± 0.12
	range	25.15–29.12	8.55–10.66	9.73–11.11	4.85–7.70	1.92–2.31	2.68–3.05	6.89–8.03	5.65–6.75	15.11–17.97	2.63–2.95
*S. v. virginiae*	*n*	7	7	7	7	7	7	7	7	7	7
	*x¯* ± *σ*	26.29 ± 1.45	9.11 ± 0.57	10.51 ± 0.64	5.77 ± 0.57	2.00 ± 0.34	2.99 ± 0.31	7.04 ± 0.53	5.55 ± 0.56	16.58 ± 1.12	2.73 ± 0.12
	range	24.37–28.13	8.31–9.70	9.47–11.27	4.89–6.46	1.53–2.46	2.45–3.45	6.43–7.81	4.93–6.57	15.26–18.32	2.53–2.91

		RWA	IFL	MFL	IFMF	IBW	ATL	ATW	OBW	MW	CW
*S. v. nitela*	*n*	19	12	12	11	16	16	16	16	18	17
	*x¯* ± *σ*	4.21 ± 0.33	2.58 ± 0.16	3.14 ± 0.23	4.40 ± 0.49	3.71 ± 0.33	3.15 ± 0.15	3.50 ± 0.21	10.40 ± 0.60	10.75 ± 0.43	6.10 ± 0.37
	range	3.79–4.89	2.33–2.86	2.62–3.46	3.45–5.28	3.22–4.53	2.90–3.42	3.10–3.73	9.60–11.52	10.02–11.53	5.44–6.83
*S. v. rufigenis*	*n*	7	7	6	7	7	7	7	7	7	7
	*x¯* ± *σ*	4.83 ± 0.3	3.12 ± 0.36	3.4 ± 0.08	4.52 ± 0.42	4.5 ± 0.24	2.89 ± 0.11	3.4 ± 0.29	10.71 ± 0.62	11.46 ± 0.29	6.57 ± 0.48
	range	4.28–5.28	2.61–3.74	3.32–3.54	3.95–5.25	4.15–4.88	2.74–3.01	3.16–3.99	9.88–11.83	10.92–11.72	5.86–7.24
*S. v. virginiae*	*n*	7	4	3	4	7	7	7	7	7	7
	*x¯* ± *σ*	4.67 ± 0.22	2.69 ± 0.11	3.13 ± 0.06	4.79 ± 0.67	4.31 ± 0.43	3.22 ± 0.32	3.62 ± 0.27	10.89 ± 0.64	11.41 ± 0.62	6.56 ± 0.28
	range	4.44–4.95	2.54–2.80	3.08–3.19	3.92–5.32	3.61–5.05	2.83–3.57	3.23–3.90	10.14–11.75	10.56–12.32	6.11–6.88

		UCML	UML	UPL	M2W	M3W	DL	LIML	LML	LPL	DH
*S. v. nitela*	*n*	19	19	19	19	19	18	19	19	19	19
	*x¯* ± *σ*	10.06 ± 0.42	5.20 ± 0.21	3.13 ± 0.24	2.10 ± 0.09	2.09 ± 0.12	20.51 ± 1.08	11.38 ± 0.45	6.13 ± 0.17	3.15 ± 0.19	3.01 ± 0.28
	range	9.37–10.67	4.81–5.61	2.85–3.81	1.97–2.26	1.85–2.35	19.04–22.87	10.54–12.29	5.83–6.38	2.91–3.66	2.60–3.65
*S. v. rufigenis*	*n*	7	7	7	7	7	7	7	7	7	7
	*x¯* ± *σ*	10.86 ± 0.56	5.6 ± 0.09	3.44 ± 0.21	2.28 ± 0.08	2.31 ± 0.11	22.47 ± 1.3	12.64 ± 0.56	6.66 ± 0.13	3.66 ± 0.33	3.33 ± 0.44
	range	9.92–11.39	5.41–5.71	3.05–3.62	2.18–2.38	2.19–2.47	20.94–24.74	11.83–13.23	6.46–6.84	3.14–4.17	2.73–3.86
*S. v. virginiae*	*n*	7	7	7	6	6	7	7	7	7	6
	*x¯* ± *σ*	10.88 ± 0.35	5.56 ± 0.17	3.62 ± 0.14	2.28 ± 0.09	2.3 ± 0.08	21.9 ± 1.30	12.4 ± 0.35	6.44 ± 0.21	3.44 ± 0.11	3.38 ± 0.28
	range	10.43–11.42	5.34–5.89	3.45–3.78	2.15–2.36	2.19–2.37	20.23–23.42	11.87–12.89	6.03–6.68	3.27–3.59	2.99–3.71

*Note*: Number of individuals (*n*), mean and S.D. (*x¯ ± σ*), and range.

**FIGURE 5:**
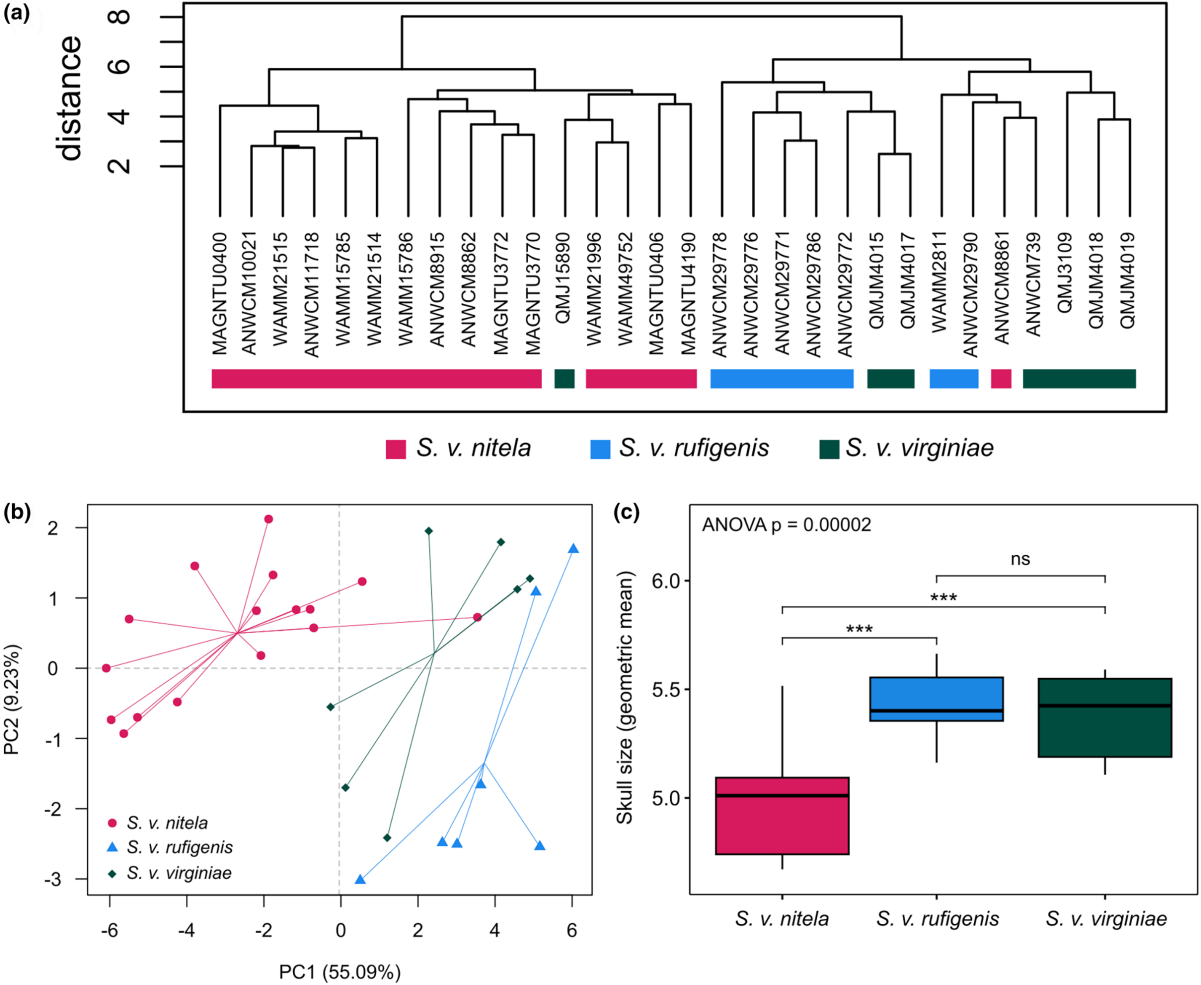
(a) Hierarchical clustering dendrogram of Euclidean distance in cranial measurements between individual *Sminthopsis virginiae* specimens measured in this study. (b) Principal Components Analysis of cranial measurements for the three subspecies. (c) Comparison of skull size (as the geometric mean) for each of the three subspecies, *p* values <.001 = ***; ns >0.05.

**FIGURE A4:**
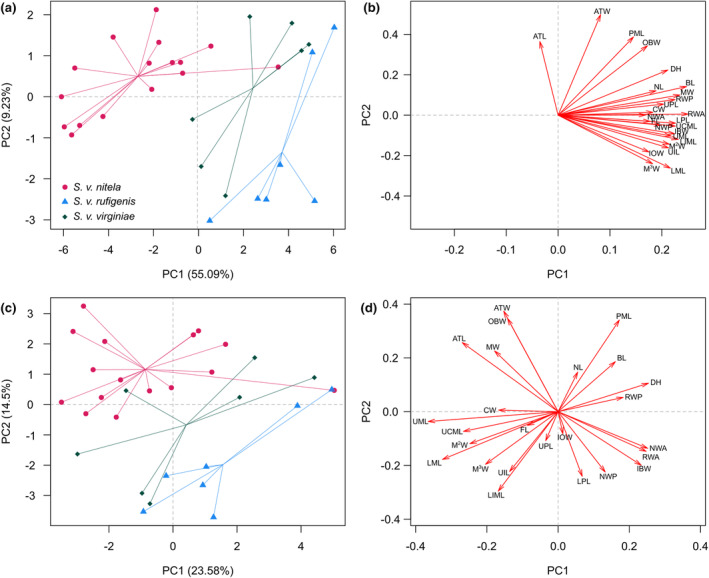
(a) PCA on *Sminthopsis virginiae* raw data with (b) contributions of each variable to the ordination. (c) PCA on size‐corrected data with (d) contribution of size‐corrected measurements to the ordination.

Hierarchical clustering analysis sorted the samples into two clusters, one being most *Sminthopsis v. nitela* together with one *S. v. virginiae* (QM JM15890), and the other cluster comprising all *S. v. rufigenis*, most *S. v. virginiae* and a single *S. v. nitela* individual (ANWC M8861) (see Figure 5a). Principal Components Analysis (PCA) showed over half of the variation in the data (55%) was explained by PC1 with PC2 explaining 9% (Figure 5b); 70% of the variation in the data was explained by only the first three PCs. Almost all measurement variables overwhelmingly contributed to PC1 and indicated size, with greater PC1 scores = larger skull size. PC2 indicated the length and width of the auditory bullae measurements (Figure A4). Analysis on skull size (as the geometric mean) also showed that *S. v. nitela* is significantly smaller than the other two subspecies (ANOVA *p*‐value = .00002), and *S. v. rufigenis* and *S. v. virginiae* could not be separated by size (*p*‐value = .84) (Figure 5c).

Removing size as a variable from the remaining data and re‐running the PCA did not improve the separation of the three subspecies in ordinal space, but 70% of the variation in the data was explained by the first 6 PCs with variables contributing more evenly to the ordinal space (Figure A4). K‐nearest neighbor discriminant analysis was slightly more successful at correctly classifying individuals to subspecies in the raw data (80% correct) than the dataset with size removed (76.7% correct), but it performed better at classifying the *Sminthopsis v. virginiae* samples when size was removed (Table 5). Overall, the analysis performed better when individuals were grouped according to the genetic clades, “*nitela*” versus “*rufigenis*” + “*virginiae*” with the raw data (93.3% correct). See Table A5 for the exact numbers of individuals classified into each category.

**TABLE 5:** Percent of specimens correctly classified into subspecies or genetic clade using k‐nearest neighbor discriminant analysis on the raw measurement data and the size‐corrected data.

Subspecies	*n*	% correct raw data	% correct size‐corrected	Genetic clade	*n*	% correct raw data	% correct size‐corrected
*nitela*	16	93.8	87.5	*nitela*	16	93.8	87.5
*rufigenis*	7	85.7	57.1	*rufigenis* + *virginiae*	14	92.9	78.6
*virginiae*	7	42.9	71.4				
Total	30	80.0	76.7	Total	30	93.3	83.3

### 3.6 Qualitative cranial examinations

Archer (1981) proposed that *Sminthopsis v. nitela* could be distinguished from *S. v. rufigenis* and *S. v. virginiae* on size and the absence of an anterior cingulum on the upper molars. We examined specimens for the presence of an anterior cingulum as well as the presence, shape, and degree of development of other molar tooth cusps. We found an anterior cingulum to be present on all upper molars (M^1–4^) in all individuals from the three subspecies (except WAMM4056), but the size differed. In *S. v. nitela*, the anterior cingulum on M^1^ tended to be reduced compared to *S. v. rufigenis* and *S. v. virginiae*. An anterior cingulum was present on M^2–4^ for all specimens examined. However, we observed several other characters that more consistently differed between the subspecies. The protoconule on M^1^ tended to be well‐formed in *S. v. rufigenis* and *S. v. virginiae*, whereas it was reduced in *S. v. nitela*. Where visible in the absence of dental wear, the stylar cusp B on M^1^ varied in size and connected to the paracrista in *S. v. nitela*, but it was always large and did not connect to the paracrista in *S. v. rufigenis* and *S. v. virginiae*. The entoconid on M_3_ tended to be larger in *S. v. rufigenis* and *S. v. virginiae* when compared to *S. v. nitela*. The posthypocristid on M_4_ was absent in all *S. v. nitela* specimens examined, whereas it was present and well‐defined in *S. v. rufigenis* and *S. v. virginiae*, where visible in the absence of tooth wear. A postorbital process was absent from all *S. v. nitela* specimens except ANWCM29861 and ANWCM8861, whereas it was present in almost all *S. v. rufigenis* and *S. v. virginiae* examined.

### 3.7 External morphology

**FIGURE 6:**
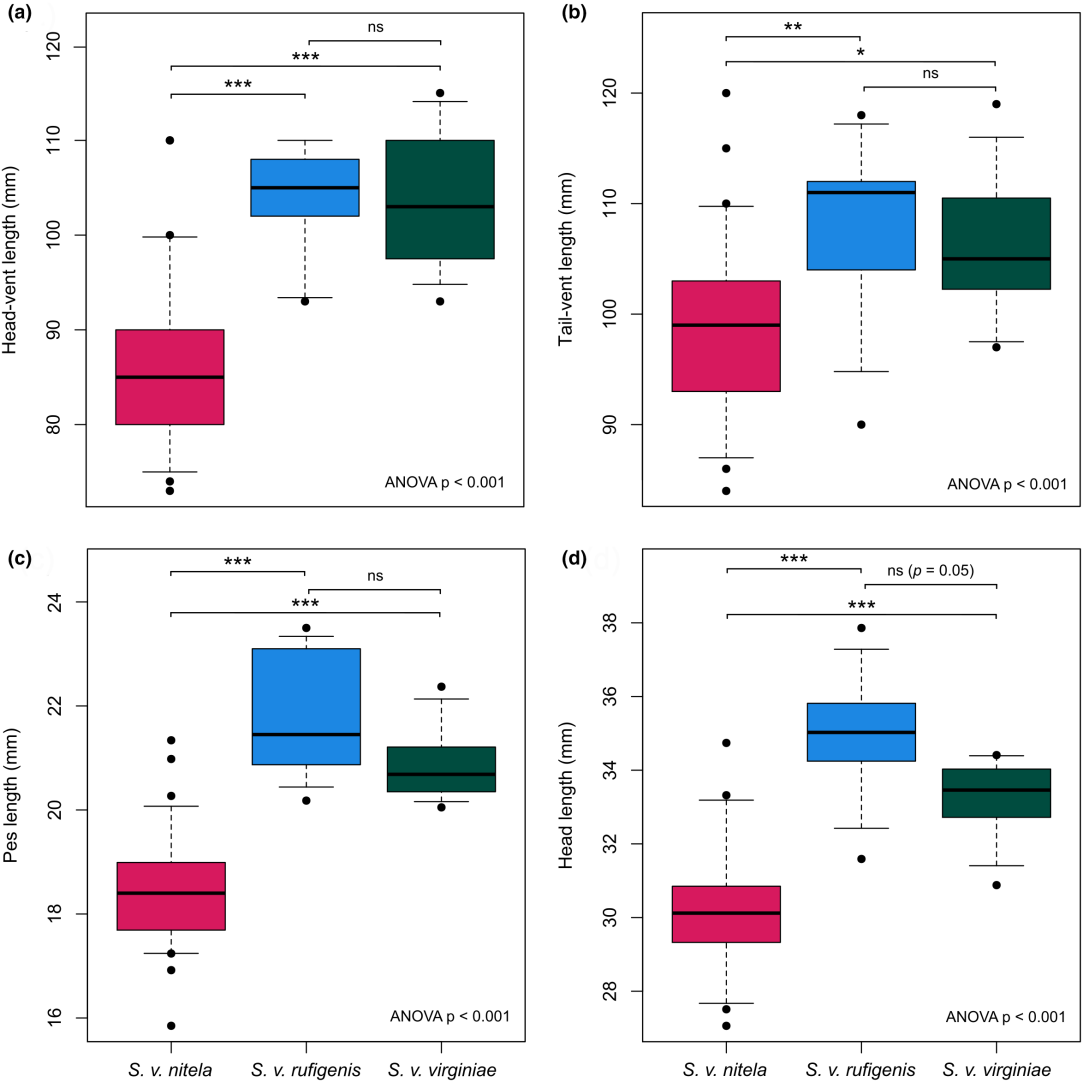
Boxplots of external measurements of *Sminthopsis virginiae* subspecies where ANOVA *p*‐values were significant, (a) head‐vent length, (b) tail‐vent length, (c) pes length, and (d) head length. *p*‐values for pairwise comparisons between subspecies are shown above plot data, ns = not significant, that is, *p* > .05, * = *p* < .05, ** = *p* < .01, and *** = *p* < .001.

**FIGURE 7:**
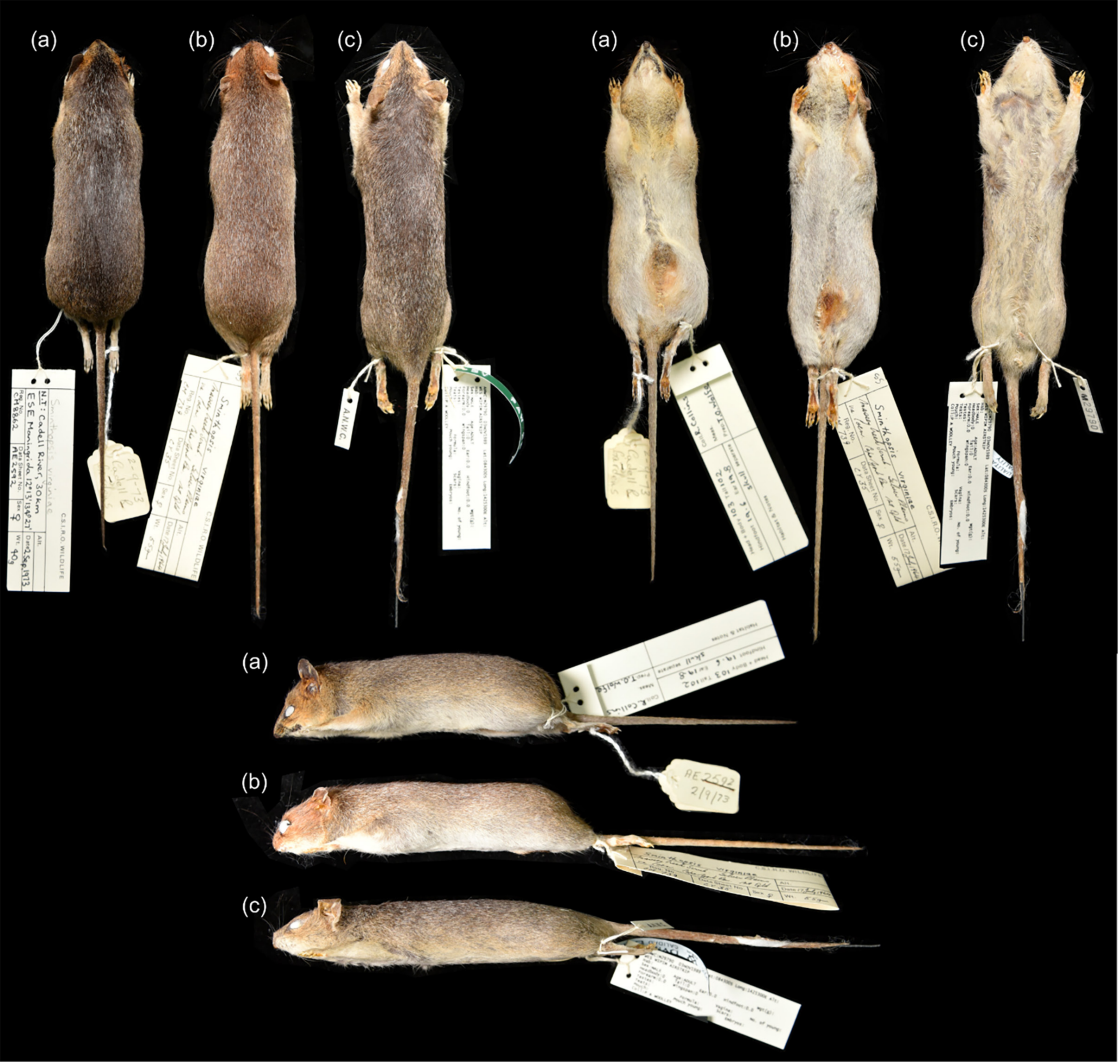
Photographs of dry preserved specimens of *Sminthopsis virginiae* representing the three subspecies, (a) ANWCM8862 *S. virginiae nitela*, (b) ANWCM739 *S. v. virginiae*, and (c) ANWCM29790 *S. v. rufigenis*. Specimens all photographed on the same scale (not shown).

As with the cranial dataset, *Sminthopsis v. nitela* was found to be significantly smaller than both *S. v. rufigenis* and *S. v. virginiae* for all measurements (Figure 6, Table 6), whereas *S. v. rufigenis* and *S. v. virginiae* could not be distinguished based on size. Individuals from New Guinea (*S. v. rufigenis*) had the largest pes (hindfoot) and head length, two variables less prone to distortion on wet preserved specimens, but this was not significantly different to individuals from Queensland. Examination of dry study skins from each subspecies (*S. v. nitela* = 9, *S. v. rufigenis* = 3, and *S. v. virginiae* = 1) showed no obvious or unique differences in pelage color, tail thickness, or footpad morphology (Figure 7). Slight variations in the depth and spread of the facial russet coloration and the width and continuity of the central facial stripe were observed across specimens both within and between subspecies. Variation in the robustness of individuals was observed across the species distribution, with the stockiest individuals being from PNG and Qld, with the NT also having some large, robust males. In contrast, most individuals from WA were smaller, with more gracile snouts.

**TABLE 6:** Summary of external measurements of *Sminthopsis virginiae* specimens measured in this study (except for weights which were taken from museum databases). Measurements are given as mean ± standard deviation ($\bar{x}\pm \sigma$) and in mm and weights in grams.

Subspecies	Sex		Head‐vent length	Tail‐vent length	Pes length	Ear length	Head length	Weight
*S. v. nitela*	♂♂	*n*	17	20	21	18	18	17
		$\bar{x}\pm \sigma$	85.5 ± 8.9	99.4 ± 9.1	18.76 ± 1.04	18.46 ± 2.25	30.41 ± 1.68	21.96 ± 7.99
		range	73–110	84–120	17.25–21.34	13.67–23.03	27.06–34.74	10.5–45
	♀♀	*n*	25	26	26	23	22	20
		$\bar{x}\pm \sigma$	85.9 ± 8.3	96.7 ± 6.0	18.03 ± 0.81	18.91 ± 1.28	29.95 ± 1.34	21.10 ± 6.82
		range	75–110	86–105	15.85–19.52	16.43–21.72	27.51–33.18	12.5–40
*S. v. rufigenis*	♂♂	*n*	5	5	6	6	4	‐
		$\bar{x}\pm \sigma$	104.8 ± 6.8	111.8 ± 5.60	22.12 ± 1.25	20.48 ± 1.83	36.03 ± 1.36	‐
		range	93–110	104–118	20.76–23.50	18.50–22.94	34.79–37.86	‐
	♀♀	*n*	4	4	4	4	4	‐
		$\bar{x}\pm \sigma$	102.5 ± 6.6	103.8 ± 10.21	21.15 ± 078	19.27 ± 1.56	33.88 ± 1.64	‐
		range	94–110	90–112	20.18–21.80	17.60–21.23	31.59–35.42	‐
*S. v. virginiae*	♂♂	*n*	4	4	5	5	4	‐
		$\bar{x}\pm \sigma$	104.1 ± 6.5	103.8 ± 5.1	21.37 ± 0.76	19.86 ± 1.59	33.77 ± 0.43	‐
		range	97.5–113	98–110	20.6–22.37	18.13–22.23	33.40–34.37	‐
	♀♀	*n*	6	7	7	7	6	3
		$\bar{x}\pm \sigma$	103.8 ± 8.1	107.4 ± 7.4	20.53 ± 0.43	19.13 ± 1.09	32.88 ± 1.30	33.50 ± 17.08
		range	93–115	97–119	20.05–21.26	17.53–20.95	30.88–34.41	18–55

*Note*: No/very little data was available for weights from *S. v. rufigenis* and *S. v. virginiae*.

## 4 DISCUSSION

In this study, we have undertaken the first combined morphological and genetic review of the variation within the tropical dasyurid marsupial species, *Sminthopsis virginiae*. Building on the work of Blacket et al. (2001) and Archer (1981) we have examined and sequenced multiple exemplars from across the species' distribution to comprehensively assess the diversity within the taxon. Our results consistently resolved the species into two morphologically and genetically distinct clades: one comprising *S. v. nitela* individuals from Western Australia and the Northern Territory, the other comprising exemplars of both *S. v. virginiae* (Queensland) and *S. v. rufigenis* (Papua New Guinea).

### 4.1 Molecular divergence in *Sminthopsis virginiae*

Using allozyme loci, Blacket et al. (2001) found fixed differences between subspecies “*nitela*” (*n* = 10) and *“rufigenis*” (*n* = 6), but these were less clear‐cut and using a much smaller sample than that sequenced here. Using additional mitochondrial and nuclear gene loci, we have corroborated the genetic distinctness of *S. v. nitela* from the other two currently recognized subspecies of *S. virginiae*. In all of our phylogenetic analyses in which individual genetic loci were weighted appropriately, or in which 3rd codon positions of protein‐coding genes were weighted and the mitochondrial 12S rRNA locus was partitioned as Stems and Loops, the resulting tree always resolved two distinct clades: one comprising all Northern Territory and Western Australian specimens (“*nitela*”) and one comprising the Queensland (“*virginiae*”) + Papua New Guinean (“*rufigenis*”) specimens, of which the latter two subspecies were intermingled rather than forming two distinct lineages. Our results suggest that although Queensland and Trans‐Fly Plains (PNG) populations of *S. virginiae* have been separated from one another by the formation of Torres Strait ~9000 years ago, genetically they are indistinguishable and are not monophyletic entities. In contrast, the user‐tree positing genetic differences between NT and WA samples of “*nitela*” was not significantly different from the best tree (Table 1).

The multiple exemplars of the three currently recognized subspecies in our data thus suggest a marked genetic divergence between *Sminthopsis v. nitela* (Kimberley and Northern Territory) and *S. v. virginiae* (Queensland)/*S. v. rufigenis* (Papua New Guinea). These “western” and “eastern” geographic groupings are separated from one another by the Carpentaria Barrier or Basin (Edwards et al., 2017), an area of unsuitable habitat for multiple species in northern Australia and a well‐known biogeographic barrier (Catullo et al., 2014; Cremona et al., 2021; Eldridge et al., 2014; von Takach et al., 2022). Due to the mitochondrial divergence (~8.2% K2P differences) reported here, we hypothesize that *S. v. nitela* diverged from their Cape York (Qld) and PNG conspecifics ~3.9 million years ago (ma) in the early‐mid Pliocene, assuming a rate of 2.1% divergence per million years for dasyurids at cytb (Krajewski et al., 2000). These estimates are similar to those reported for *Melithreptus* honeyeaters (Toon et al., 2010) and the common wallaroo (Eldridge et al., 2014), but are much older than divergences between species of *Petaurus* gliders (87–405 thousand years ago, Cremona et al., 2021) and the antillopine wallaroo, which exhibited some gene flow across the barrier (Eldridge et al., 2014).

We acknowledge that divergence estimates calculated on single locus data may not be reflective of the true population divergence time and that it is difficult to obtain robust divergence times for Sminthopsins due to a lack of suitable fossils available in this group (Krajewski et al., 2024). The rate we have used for cytb assumes a divergence between thylacines and dasyurids of 25 mya; however, more recent studies have estimated that divergence occurred ~40 mya (Mitchell et al., 2014; Westerman et al., 2016), which suggests our divergence values are conservative.

We noted much smaller genetic differences between *Sminthopsis v. nitela* populations from the Kimberley and those from the Northern Territory, suggesting a much more recent divergence (0.67 ma; <1.3%), which is separated by the Ord Basin, an area long recognized as being a biogeographic boundary for vertebrate species occurring across the Kimberley and Top End of the NT (Catullo et al., 2014; Edwards et al., 2017; Potter et al., 2012).

Importantly, although the degree of cytb K2P distances between *Sminthopsis v. nitela* and *S. v. virginiae* (Qld)/*S. v rufigenis* (PNG) is only about half of the values calculated between some other currently recognized species in the *Sminthopsis* “*macroura*” species group (~8.62% cf 14%–20%, see Table 2) they are nevertheless comparable with K2P values reported between currently recognized species in the *Sminthopsis* “*murina*” group. These values range from 7.5% (*S. murina* v *S. gilberti*) to 18.6% (*S. murina* v *S. hirtipes*) (MW, unpublished data).

### 4.2 Connection across the Torres Strait

In contrast to the marked genetic differences observed between western “*nitela”* and eastern “*virginiae* + *rufigenis”* samples, only minimal differences (~1.2%, see Table 2) were noted between specimens of the currently recognized subspecies *Sminthopsis v. virginiae* from the Cape York Peninsula (CY) and *S. v. rufigenis* from the Trans‐Fly region of Papua New Guinea. This suggests that the genetic differences between these two genetic lineages are not much greater than the differences observed between individuals within each of the two subspecies. Most likely, these two taxa, currently separated by the Torres Strait, have not long diverged from one another, or there has been gene flow between the two populations during periods when the two regions were contiguous at times of lower sea levels (Voris, 2000).

Glacial cycles during the Pleistocene caused periodic exposure of land surfaces on the Sahul Shelf between Australia and New Guinea that were covered by savanna woodland including Lake Carpentaria (Figure 1) (Reeves et al., 2008; Torgersen et al., 1983). Such habitats would have potentially allowed periodic exchange (and isolation of populations) of terrestrial fauna favoring savannah and woodland habitats, including *Sminthopsis virginiae*, between the two land masses (Lavery & Leung, 2023; Wüster et al., 2005). The Sahul Shelf was progressively inundated due to sea‐level rise after the last glacial maximum and a final land bridge between Australia and New Guinea was last cut about 9000 years ago with the formation of the Torres Strait, thus preventing further genetic exchange between the populations isolated on one or other of the land masses (Voris, 2000).

### 4.3 Morphological divergence in *Sminthopsis virginiae*

Our comprehensive morphological analyses strongly accord with the genetic data in grouping western “*nitela*” as divergent from a combined grouping of eastern “*virginiae*” + northern “*rufigenis*”.

In his major morphological revision of *Sminthopsis*, Archer (1981) recognized three subspecies of *S. virginiae* corresponding to isolated populations across northern Australia and southern Papua New Guinea (Figure 1). Archer (1981) stated that *S. v. nitela* was morphologically distinguishable from the other two subspecies in being smaller and in lacking a continuous anterior cingulum on the upper molar teeth. Our results concord with Archer (1981) in that *S. v. nitela* is indeed smaller than *S. v. rufigenis* and *S. v. virginiae*; however, with our larger sample, we observed substantial variation in the presence and development of an anterior cingulum on the upper molar teeth. This feature is in fact present in all three subspecies on all upper molars (M^1–4^), but varies between them, being reduced on M^1^ in *S. v. nitela*. However, we found that *S. v. nitela* could be clearly distinguished from *S. v. rufigenis* and *S. v. virginiae* based on various cranial and external measurements, but that *S. v. rufigenis* and *S. v. virginiae* could not be distinguished from each other based on the cranial features we investigated.

Archer also noted that specimens of *nitela* were “somewhat diverse and may represent more than one form” (Archer, 1981: 141). Some of these individuals have subsequently been recognized as separate species—*S. bindi* and *S. butleri*‐and other specimens examined by Archer (1981) are exemplars of *S. macroura* and another dasyurid genus, *Pseudantechinus*. In the present study, we found no indication of multiple forms of *S. v. nitela*.

### 4.4 Taxonomic implications

Little is known about the capacity of “*nitela*,” “*virginiae*,” and “*rufigenis*” individuals to interbreed and produce viable offspring. The disjunct distributions of the three subspecies (see Figure 1) suggest that hybridization would not occur naturally. Wild populations of “*nitela*” also appear to have a different reproductive strategy to that seen in “*virginiae*” and “*rufigenis*” in being seasonal breeders with reproduction occurring from July to February (Morton et al., 1987). In contrast, the Qld and PNG forms of *S. virginiae* are thought breed year round (Taplin, 1980; Woolley, 1994). We note that although Woolley (2023) reported that a male “*nitela*” was able to sire offspring when crossed *in the laboratory* with a female from Queensland (“*virginiae*”), there is no evidence that the progeny was fertile. In contrast, NT (“*nitela*”) males never produced offspring when mated with PNG (“*rufigenis*”) females (P. A. Woolley, pers. comm.).

We have demonstrated reciprocal monophyly and morphological differentiation of Western Australian and Northern Territory specimens of *Sminthopsis virginiae* from their Queensland and Papuan New Guinean conspecifics. Our genetic and morphological data strongly suggest that “*nitela”* individuals are sufficiently distinct from specimens of the currently recognized subspecies “*virginiae*” and “*rufigenis*” to warrant reinstatement as a full species under the Unified Species Concept (De Queiroz, 2007)—*Sminthopsis nitela—*as originally proposed by Collett (1897) and suggested by Blacket et al. (2001). But what of continued recognition of the currently recognized subspecies *S. virginiae virginiae* and *S. v. rufigenis*? These “lineages” are not reciprocally monophyletic in our phylogenetic analyses and differ in only a few morphological characters. However, the two subspecies are separated geographically by the Torres Strait and are unlikely to naturally come into contact. They also differ in nipple number between females (6 in *S. v. rufigenis* and 8 in *S. v. virginiae*), which is a character that often discriminates taxa within dasyurids (Baker & Dickman, 2018). Taken together, we propose the subspecific status of Queensland and Papua New Guinean *S. virginiae* specimens be retained, in the interest of recognizing the two geographically distinct populations. The taxonomic revision of *S. virginiae* is formalized in the section below.

### 4.5 Taxonomic revision

#### 4.5.1 Systematics

Family Dasyuridae Goldfuss, 1820.

Genus *Sminthopsis* Thomas, 1887.

*Sminthopsis virginiae* (de Tarragon, 1847).

*Sminthopsis virginiae virginiae* (de Tarragon, 1847).

*Sminthopsis virginiae rufigenis* Thomas, 1922.

##### Etymology

de Tarragon (1847) gave no indication of the meaning of “*virginiae*” (Archer, 1981).

##### Synonyms

*Phascogale virginiae* de Tarragon, 1847; *Sminthopsis rufigenis* Thomas, 1922; *Sminthopsis lumholtzi* Iredale & Troughton, 1934; *Phascogale rona* Tate & Archbold, 1936.

##### Type details

**FIGURE 8:**
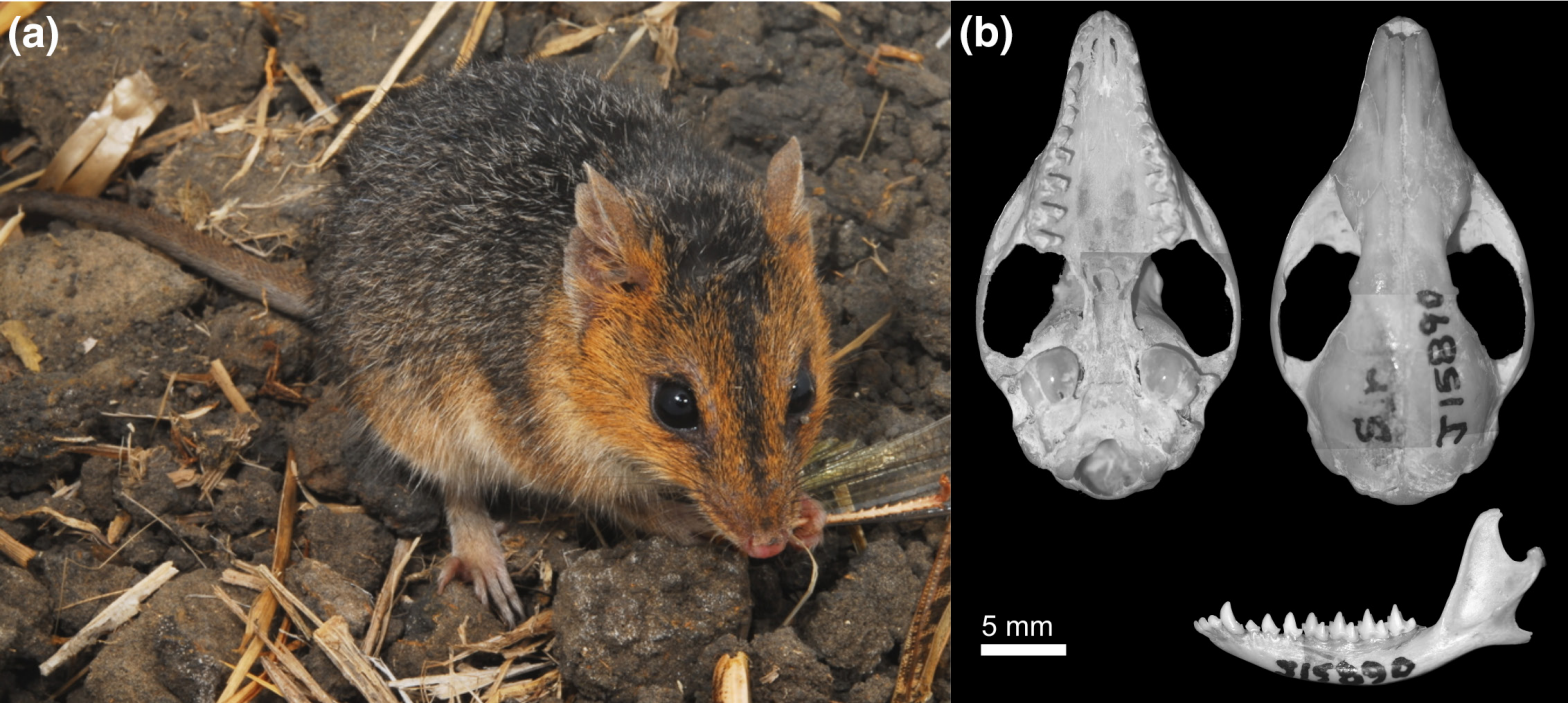
(a) *Sminthopsis virginiae* in life from Queensland, Australia (Photo: Eric Vanderduys), (b) cranium and dentary of the neotype QMJ15890.

Neotype, QMJ15890 adult male skull, and wet specimen, Herbert Vale, Queensland, designated by Archer (1981). The original type specimen described by de Tarragon is considered lost (Collett, 1887a), and no type locality was listed, but a subsequent description of the species based on a specimen collected from Herbert Vale, Queensland was published by Collett (1887a, 1887b). We have examined the neotype (see Figure 8b).

##### Remarks

Here, we include two forms under this species, *Sminthopsis v. virginiae* and *S. v. rufigenis* based on our combined molecular and morphological analysis. It differs from all other species of *Sminthopsis* by the combination of the following characters: rufous cheeks and dark facial stripe, conspicuously enlarged oval apical granules on the interdigital footpads, thin tail, large body size (although smaller than *S. douglasi* and *S. psammophila*) and the presence of continuous anterior cingula on the upper molars (see Archer, 1981). *Sminthopsis virginiae* can be distinguished from *S. nitela* by genetic divergence, larger size, and other craniodental characteristics detailed below. Both subspecies were accepted as valid taxa by Archer (1981) and subsequent authors (e.g., Jackson et al., 2015) and are considered to be aseasonal breeders (Taplin, 1980; Woolley, 1994). Females of the two subspecies can be distinguished by nipple number, six in *S. v. rufigenis* and eight in *S. v. virginiae*. A full list of material examined can be found in Table A1. Note, while we did not include genetic material from the Aru Islands in this study, this form was considered to be the same as those from Papua New Guinea by Archer (1981).

##### Geographic distribution

This species occurs in two disjunct populations separated by the Torres Strait, *S. v. rufigenis* in the Aru Islands and Trans‐fly region of New Guinea, and *S. v. virginiae* in Queensland on the Cape York Peninsula and along the north‐eastern coast of Australia (Figure 1).

##### Diagnosis

Morphological description of *Sminthopsis virginiae* is included in Collett (1887a, 1887b), and an extensive description is provided by Archer (1981). We provide a brief diagnosis. *Sminthopsis virginiae* is a large species of *Sminthopsis* with striking russet‐orange coloration to the sides of the face and a dark central stripe on the head. The tail is always longer than the head‐body length and does not become incrassated (i.e., fattened at the base) like other members of the “*macroura*” species group. Apical granules on the hindfoot pads (underside of the foot) are large and elongate, sometimes appearing striated (see Archer, 1981). *Sminthopsis virginiae* differs from *S. nitela* by larger size; a well‐developed anterior cingulum on M^1^; protoconule and a large stylar cusp B on M^1^ that does not connect to the paracrista; a larger entoconid on M_3_; and a present and well‐defined posthypocristid on M_4_. A postorbital process is often present and well developed (which is rare in *S. nitela*).

##### Recommended common names

We propose the common names as follows: eastern red‐cheeked dunnart for both *Sminthopsis virginiae* and *S. v. virginiae*, and northern red‐cheeked dunnart for *S. v. rufigenis*. These names delineate the various regional distributions of each form relative to *S. nitela* in the west, see below.

#### 4.5.2 Sminthopsis nitela Collett, 1897

##### Etymology

Collett (1897) gave no indication on the meaning behind the species name, “nitela.” In Latin, it means “brightness” or “splendor,” and this may allude to the bright orange fur patches on the sides of the face.

##### Type details

**FIGURE 9:**
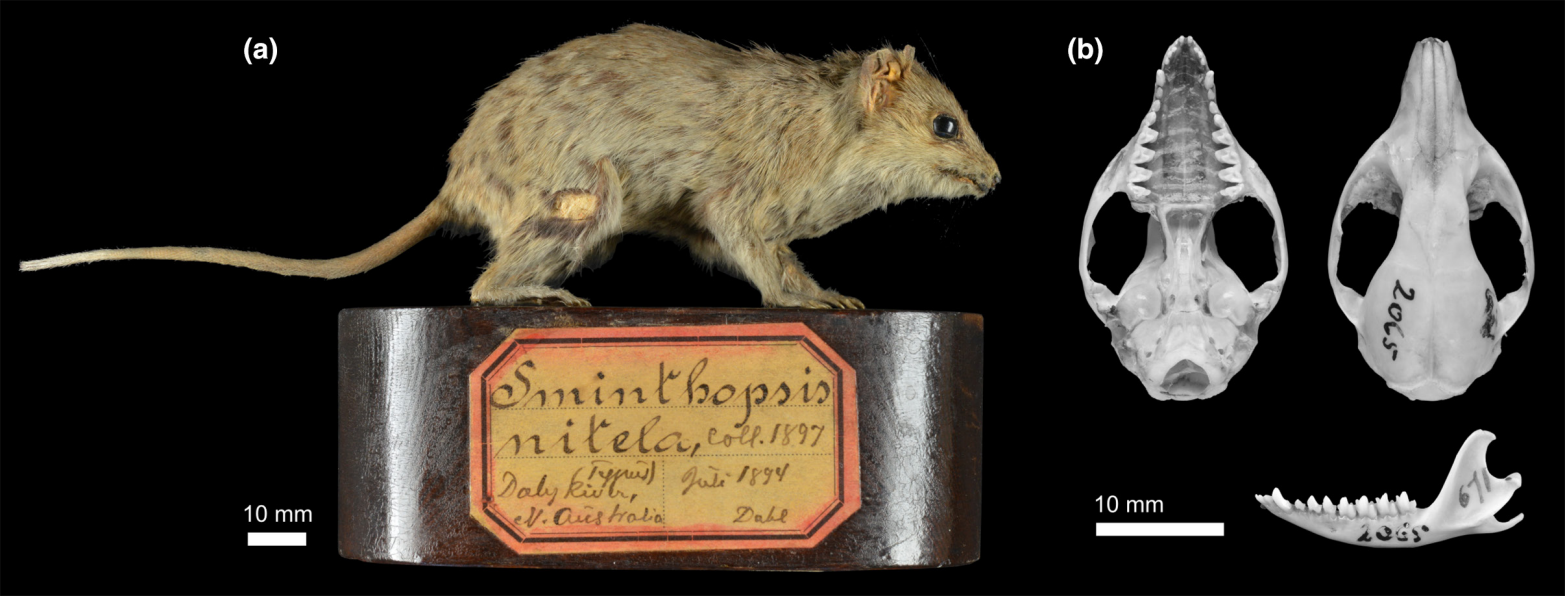
Photograph of *Sminthopsis nitela* lectotype NHMO‐DMA‐31007, (a) mounted specimen, (b) cranium and dentary. Note that fur coloration has faded in the mounted specimen. Photos: Lars Erik Johannessen, NHMO.

Collett (1897) described the species from four specimens all collected from the Daly River area, NT in July and October 1894 by Knut Dahl (Figure 1). Three of these reside in the Natural History Museum, University of Oslo, Norway (Wiig & Bachmann, 2013). Here, we designate a lectotype and paralectotypes from this series. Lectotype: NHMO‐DMA‐31007 adult male, skull and dry mounted specimen July 1894 (Figure 9). Paralectotypes: NHMO‐DMA‐55094 whole specimen in ethanol collected July 1894; NHMO‐DMA‐31006 male collected 3rd October 1894, skull out and study skin; NHMUK1897.4.12.6 skin with extracted skull, and the latter specimen was examined by Archer (1981).

##### Remarks

Differs from all other species of *Sminthopsis* in the same ways that *S. virginiae* differs. We recognize *Sminthopsis nitela* as a separately evolving lineage from *S. virginiae* (see the unified species concept of De Queiroz, 2007), from our comprehensive evidence of molecular and morphological distinctiveness, geographic isolation, and differences in breeding biology (Morton et al., 1987). *Sminthopsis nitela* differs by 8.09%–8.23% divergence at the mitochondrial cytb and 0.9%–1.6% at 12S loci from *S. virginiae*.

##### Geographic distribution

Confined to several localities in the Kimberley, WA, including Mitchell Plateau, Kalumburu, and Sir Graham Moore Island, and widespread in the Top End of the NT, including the Tiwi islands (see Figure 1).

##### Diagnosis

See description by Collett (1897) and further details in Archer (1981). Like *Sminthopsis virginiae*, but generally smaller in all aspects of external and craniodental proportions with occasional large males from the NT population reaching sizes similar to *S. v. virginiae* individuals. Differs from *S. virginiae* in possessing a reduced anterior cingulum and protoconule and a variable‐sized stylar cusp B that connects to paracrista on M^1^, with a smaller entoconid on M^3^ compared to *S. virginiae* and rarely with a postorbital process.

##### Recommended common name

We suggest the name for *S. nitela* as follows: western red‐cheeked dunnart. This delineates the regional distribution of *S. nitela* in the west from its sister species, the eastern red‐cheeked dunnart, *S. virginiae* (the latter with subspecies in Qld and PNG).

### 4.6 Conservation implications

Here we have confirmed that *Sminthopsis nitela* is restricted to the Kimberley and areas of the Northern Territory and that *S. v. virginiae* is only found in Queensland and *S. v. rufigenis* is distributed outside of Australia, in New Guinea and the Aru Islands. *Sminthopsis virginiae* was assessed under IUCN criteria in 2015 and populations were considered stable and listed as least concern (Helgen et al., 2016). None of the Australian populations are listed as threatened under the federal EPBC Act. Both red‐cheeked dunnart species are infrequently encountered, and little is known of their ecology; it is thought that their populations fluctuate with seasonal conditions (Woolley, 2023). Given ongoing declines in small mammal populations in northern Australia (Cremona et al., 2022; Woinarski et al., 2011) and the elusive nature of red‐cheeked dunnart species, it will be difficult to detect changes in the species' abundance throughout their ranges.

## AUTHOR CONTRIBUTIONS

**Linette S. Umbrello:** Conceptualization (lead); data curation (lead); formal analysis (lead); funding acquisition (lead); investigation (equal); methodology (equal); project administration (lead); supervision (lead); visualization (lead); writing – original draft (lead); writing – review and editing (lead). **Hayley Newton:** Formal analysis (equal); funding acquisition (supporting); investigation (lead); methodology (equal); visualization (supporting); writing – original draft (supporting); writing – review and editing (supporting). **Andrew M. Baker:** Conceptualization (equal); funding acquisition (equal); supervision (supporting); writing – review and editing (equal). **Kenny J. Travouillon:** Conceptualization (equal); funding acquisition (equal); investigation (supporting); supervision (supporting); writing – review and editing (supporting). **Michael Westerman:** Conceptualization (lead); formal analysis (lead); funding acquisition (equal); methodology (equal); supervision (supporting); visualization (supporting); writing – original draft (lead); writing – review and editing (equal).

## CONFLICT OF INTEREST STATEMENT

The authors declare no conflict of interest in this manuscript.

## Supporting information


File S1.

File S2.

## Data Availability

Individual genotype sequences have been deposited in NCBI GenBank (PP851402–PP851404, PP853397–PP853441, PP854425, PP858677–PP858743, PP887888–PP887945, PP898280–PP898286, and PP900178). Individual metadata, corresponding GenBank Accession numbers, and external morphological measurements can be found in Table A1 (including georeferences in decimal degrees and date of sampling event). Cranial measurement data can be found in the Supporting Information File S1 as a text file.

## APPENDIX A

**TABLE A1:** Details of specimens and tissue samples of *Sminthopsis* examined in this study including outgroup samples and GenBank accession numbers for each gene sequenced.

ID	Taxon	Locality	Lat	Lon	Date	Sex	Age	CR	12S	Cytb	w‐globin	bfib7	IRBP	Other No.
ABTC149280	*S. v. nitela*	NT; Litchfield	−13.2646	130.7272	2016‐05‐12				PP853398	PP887890	PP858736			CCM6438
ABTC28415	*S. v. nitela*	NT; Plumtree Creek	−12.6667	132.2500				PP858677	PP853399	PP887888	PP858718	PP887931	PP898280	
ANWCM10021	*S. v. nitela*	NT; Mt Tolmer	−13.2167	130.7333	1970‐01‐01	M	A							
ANWCM11718	*S. v. nitela*	NT; Blyth River	−12.0667	134.5833	1975‐07‐01	F	A							
ANWCM24722	*S. v. nitela*	NT; Nourlangie Camp	−12.7583	132.6567	1981‐03‐08	F	A							
ANWCM29853	*S. v. nitela*	NT; Coomalie Farm	−13.0667	131.0333	1992‐08‐01	F	A	PP858679	PP853416	PP887910	PP858737	PP887937		LTU F24/MW597
ANWCM29861	*S. v. nitela*	NT; Donydi	−12.89	135.45	1980‐06‐19	M	A							
ANWCM8861	*S. v. nitela*	NT; Cadell River	−12.2167	134.4500	1973‐09‐02	M	A							
ANWCM8862	*S. v. nitela*	NT; Cadell River	−12.2167	134.4500	1973‐09‐02	F	A							
ANWCM8915	*S. v. nitela*	NT; Berry Springs	−12.7000	130.9667	1974‐07‐26	F	A							
MAGNTS1283	*S. v. nitela*	NT; Nitmiluk NP	−14.1112	132.5676	2015‐05‐03				PP853420					
MAGNTU0357	*S. v. nitela*	NT; Finniss River	−12.8869	130.6029	1987‐05‐15	F	A							
MAGNTU0400	*S. v. nitela*	NT; Katherine Gorge NP	−14.1167	132.5833	1981‐06‐17	M	A							
MAGNTU0406	*S. v. nitela*	NT; Coonawarra	−12.4333	130.9333	1982‐05‐11	M	A							
MAGNTU0529	*S. v. nitela*	NT; Harrison Dam	−12.5868	131.3698	1989‐01‐04	F	A							
MAGNTU2034	*S. v. nitela*	NT; Tortilla Flats	−13.0333	131.2500		M	A							
MAGNTU3769	*S. v. nitela*	NT; Wildman River	−12.6333	132.2000	1968‐07‐30	F	A							
MAGNTU3770	*S. v. nitela*	NT; Berry Springs	−12.7000	130.9667	1974‐08‐20		A							
MAGNTU3771	*S. v. nitela*	NT; Ja Ja Billabong	−12.5201	132.9369	1981‐09‐10	F	A							
MAGNTU3772	*S. v. nitela*	NT; Berry Springs	−12.7000	130.9667	1979‐01‐22	F	A							
MAGNTU4129	*S. v. nitela*	NT; Jabiluka	−12.5535	132.9536	1992‐06‐20	M	A	AF088954	PP853421	PP887914	PP858722			ABTC29157
MAGNTU4190	*S. v. nitela*	NT; Kakadu NP	−13.091	132.393		M	A							
MAGNTU4325	*S. v. nitela*	NT; Litchfield NP	−13.4037	130.9197	1996‐03‐04	M	A	PP858695	PP853422	PP887915	PP858739			ABTC29717
MAGNTU4855	*S. v. nitela*	NT; Douglas River	−13.7538	131.4532	1998‐06‐22	F	A							
MAGNTU5136	*S. v. nitela*	NT; Fogg Dam	−12.6202	131.3198	2000‐06‐27	M	A							
MAGNTU5219	*S. v. nitela*	NT; Blackmore River	−12.7369	130.9530	2001‐10‐09	F	A							
MAGNTU5353	*S. v. nitela*	NT; Fish River	−14.1873	130.9197	2001‐09‐05	F	A							
MAGNTU5877	*S. v. nitela*	NT; Kowandi	−12.5201	131.5253	2007‐06‐11	F	A							
MAGNTU5878	*S. v. nitela*	NT; Kowandi	−12.5201	131.5253	2007‐06‐11	M	A	PP858681	PP853423	PP887916				
NMVC26328	*S. v. nitela*	NT; Jabiru	−13.4119	130.8967		M		PP858680	PP853424	PP887917	PP858706	PP887938		LTU M3 NMVZ32665 ABTC51436
NMVC26329	*S. v. nitela*	NT; Jabiru	−13.4119	130.8967		M		PP858678	AF088977	PP887918	PP858717	PP887939		LTU M2 ABTC51433
WAMM25796	*S. v. nitela*	NT; Holmes Jungle	−12.4500	130.8333	1986‐08‐08	F	A							
WAMM4056	*S. v. nitela*	NT; Oenpelli	−12.3166	133.0500		M	A							
8tZ2UL^a^	*S. v. nitela*	WA; Doongan Stn.	−15.2428	126.4346	2022‐05‐25	M			PP853397					
BfQFo9^a^	*S. v. nitela*	WA; Doongan Stn.	−15.2160	126.4231	2022‐05‐25	M		PP858688		PP887912				
cNlqf8^a^	*S. v. nitela*	WA; Doongan Stn.	−15.24	126.43	2022‐05‐26	F			PP853418					
DBCAIRSV	*S. v. nitela*	WA; Laterite Cons. Park	−14.9583	125.9640	2012‐07‐01			PP858690	PP853419	PP887913	PP858713			LCI29‐2
u4dOnb^a^	*S. v. nitela*	WA; Theda Stn.	−14.8709	126.3391	2022‐05‐06	F			PP853427	PP887921				
WAMM15785	*S. v. nitela*	WA; Mitchell Plateau	−14.6694	125.7389	1976‐10‐27	F	A							
WAMM15786	*S. v. nitela*	WA; Mitchell Plateau	−14.6722	125.7361	1976‐10‐20	F	A							
WAMM21503	*S. v. nitela*	WA; Mitchell Plateau	−14.8222	125.8417	1981‐06‐19	M	SA	PP858689	PP853428					ABTC7712
WAMM21514	*S. v. nitela*	WA; Mitchell Plateau	−14.8791	125.8083	1981‐06‐26	F	A							
WAMM21515	*S. v. nitela*	WA; Mitchell Plateau	−14.8222	125.8417	1981‐06‐26	M	A	PP858685	PP853429		PP858714			ABTC7713
WAMM21601	*S. v. nitela*	WA; Mitchell Plateau	−14.7792	125.8208	1981‐10‐19	F	A	AF288576	AF339109	PP887922	PP858720			ABTC7716
WAMM21771	*S. v. nitela*	WA; Mitchell Plateau	−14.8208	125.8375	1982‐04‐11	F	A	PP858691	PP853430					ABTC7717
WAMM21772	*S. v. nitela*	WA; Mitchell Plateau	−14.8208	125.8375	1982‐04‐12	F	SA							
WAMM21789	*S. v. nitela*	WA; Mitchell Plateau	−14.8550	125.8300	1982‐04‐19	M	A							
WAMM21861	*S. v. nitela*	WA; Mitchell Plateau	−14.8722	125.8208	1982‐05‐01	M	A	PP858684	PP853431					ABTC7719
WAMM21883	*S. v. nitela*	WA; Mitchell Plateau	−14.8722	125.8208	1982‐05‐03	F	SA							
WAMM21893	*S. v. nitela*	WA; Mitchell Plateau	−14.8722	125.8208	1982‐05‐04	M	A							
WAMM21901	*S. v. nitela*	WA; Mitchell Plateau	−14.8722	125.8208	1982‐07‐10	M	A							
WAMM21910	*S. v. nitela*	WA; Mitchell Plateau	−14.8722	125.8208	1982‐07‐11	F	A							
WAMM21911	*S. v. nitela*	WA; Mitchell Plateau	−14.8722	125.8208	1982‐07‐11	F	A							
WAMM21925	*S. v. nitela*	WA; Mitchell Plateau	−14.8722	125.8208	1982‐07‐14	M	A	PP858683	PP853432		PP858721			ABTC7725
WAMM21926	*S. v. nitela*	WA; Mitchell Plateau	−14.8222	125.8417	1982‐07‐15	M	A							
WAMM21988	*S. v. nitela*	WA; Mitchell Plateau	−14.8167	125.8403	1982‐07‐24	F	A	AF288577	AF339110	PP887923	PP858715			ABTC7728
WAMM21989	*S. v. nitela*	WA; Mitchell Plateau	−14.7792	125.8208	1982‐07‐24	F	A							
WAMM21996	*S. v. nitela*	WA; Mitchell Plateau	−14.8167	125.8403	1982‐07‐24	M	A							
WAMM22067	*S. v. nitela*	WA; Mitchell Plateau	−14.8722	125.8208	1982‐09‐25	F	A	PP858682	PP853433	PP887924	PP858734	PP887941	PP898283	
WAMM22068	*S. v. nitela*	WA; Mitchell Plateau	−14.8722	125.8208	1982‐09‐24	F	A	PP858686	PP854425	PP887925	PP858733	PP887942		
WAMM49752	*S. v. nitela*	WA; Mitchell Plateau	−14.8072	125.8417	2002‐06‐21	M	A	PP858687	PP853434	PP887926	PP858738			
WAMM52669	*S. v. nitela*	WA; Mitchell Plateau	−14.6731	125.7331	2003‐05‐30	F	SA		PP853435			PP887943		
WAMM52670	*S. v. nitela*	WA; Surveyor's Pool	−14.6731	125.7331	2003‐05‐30	F	SA	PP858693	PP853436	PP887927	PP858735			
WAMM52676	*S. v. nitela*	WA; Surveyor's Pool	−14.6731	125.7331	2003‐05‐31	F	A					PP887944		
WAMM52678	*S. v. nitela*	WA; Surveyor's Pool	−14.6731	125.7331	2003‐06‐01	M	A							
WAMM54217	*S. v. nitela*	WA; Mitchell Plateau	−14.8086	125.7728	2003‐05‐16	M	A							
WAMM54888	*S. v. nitela*	WA; Sir Graham Moore Is.	−13.8717	126.5144	2007‐08‐05	F	A		PP853437	PP887928	PP858716			
WAMM54891	*S. v. nitela*	WA; Sir Graham Moore Is.	−13.8717	126.5144	2007‐08‐07	F	A		PP853438	PP887929	PP858719	PP887945		
WAMM54892	*S. v. nitela*	WA; Sir Graham Moore Is.	−13.8717	126.5144	2007‐08‐07	F	A		PP853439					
WAMM60906	*S. v. nitela*	WA; Mitchell River NP	−14.8361	125.7373	2007‐06‐19	M	A							
WAMM60929	*S. v. nitela*	WA; Mitchell River NP	−14.8361	125.7373	2007‐06‐17	M	A		PP853440					
WAMM60930	*S. v. nitela*	WA; Mitchell River NP	−14.8361	125.7373	2007‐06‐18	M	A	PP858692	PP851402					
ymaMIG^a^	*S. v. nitela*	WA; Doongan Stn.	−15.2241	126.4299	2022‐05‐27	M		PP858694	PP853441	PP887930	PP898284			
ABTC51490	*S. v. rufigenis*	PNG; Oriomo	−8.8666	143.1833		M	A	AF088953	PP853400	PP887889	PP858723	PP887932		LTU M23
ANWCM29771	*S. v. rufigenis*	PNG; Bensbach	−8.8333	141.2500	1983‐01‐16	F	A							PAW1
ANWCM29772	*S. v. rufigenis*	PNG; Bensbach	−8.8333	141.2500	1984‐05‐25	F	A							PAW2
ANWCM29773	*S. v. rufigenis*	PNG; Tupi Heathland	−8.7167	143.1500	1984‐05‐25	F	A		PP853405	PP887892				LTU F3 PAW3
ANWCM29775	*S. v. rufigenis*	PNG; Tupi Heathland	−8.7167	143.1500	1989‐10‐07	F	SA							PAW5
ANWCM29776	*S. v. rufigenis*	PNG; Oriomo	−8.8000	143.1833	1989‐10‐12	M	A							PAW6
ANWCM29777	*S. v. rufigenis*	PNG; Tupi Heathland	−8.7167	143.1500	1989‐10‐13	F	A	PP858699	PP853401	PP887893	PP858729	PP887933		LTU F7 PAW7
ANWCM29778	*S. v. rufigenis*	PNG; Tupi Heathland	−8.7167	143.1500	1989‐10‐15	F	A							PAW8
ANWCM29779	*S. v. rufigenis*	PNG; Tupi Heathland	−8.7167	143.1500	1989‐10‐17	M	A	PP858701	PP853406	PP887894	PP858741			LTU M9 PAW9
ANWCM29780	*S. v. rufigenis*	PNG; Wipim airstrip	−8.7167	142.8833	1989‐10‐27	F	A		PP853407	PP887895	PP858742			LTU F10 PAW10
ANWCM29781	*S. v. rufigenis*	PNG; Wipim airstrip	−8.7167	142.8833	1989‐10‐27	M		PP858703	PP853408	PP887896	PP858710			LTU M11 PAW11
ANWCM29782	*S. v. rufigenis*	PNG; Wipim airstrip	−8.7167	142.8833	1989‐10‐28	F	A		PP851403	PP887897	PP898285			LTU F12 PAW12
ANWCM29783	*S. v. rufigenis*	PNG; Wipim airstrip	−8.7167	142.8833	1989‐10‐28	M			PP853409	PP887898				LTU M13 PAW13
ANWCM29784	*S. v. rufigenis*	PNG; Wipim airstrip	−8.7167	142.8833	1989‐10‐29	M	A							PAW14
ANWCM29785	*S. v. rufigenis*	PNG; Wipim airstrip	−8.7167	142.8833	1989‐10‐29	M	A			PP887899	PP858730			LTU M15 PAW15
ANWCM29786	*S. v. rufigenis*	PNG; Wipim airstrip	−8.7167	142.8833	1989‐10‐31	M	A							PAW16
ANWCM29787	*S. v. rufigenis*	PNG; Wipim airstrip	−8.7167	142.8833	1989‐10‐31	M		PP858705	PP853410	PP887900	PP858708	PP887936	PP898281	LTU M17 ABTC51488
ANWCM29788	*S. v. rufigenis*	PNG; Wipim airstrip	−8.7167	142.8833	1989‐11‐01	M			PP853411	PP887901	PP858731			LTU M18 PAW18
ANWCM29789	*S. v. rufigenis*	PNG; Wipim airstrip	−8.7167	142.8833	1989‐11‐02	M	A							PAW19
ANWCM29790	*S. v. rufigenis*	PNG; Wipim village	−8.7167	142.8833	1989‐11‐03	M	A							PAW20
ANWCM29791	*S. v. rufigenis*	PNG; Wipim airstrip	−8.7167	142.8833	1989‐11‐03	F	A							PAW21
ANWCM29801	*S. v. rufigenis*	PNG; Tupi Heathland	−8.7167	143.1833		F	J		PP853412	PP887902	PP858725			LTU F31 PAW31
ANWCM29808	*S. v. rufigenis*	PNG; Tupi Heathland	−8.7167	143.1833	1989‐10‐15	M	J	PP858702	AF088976	AF088934	PP858743			LTU M38 ABTC51492
ANWCM29813	*S. v. rufigenis*	PNG; Captive bred			1990‐11‐05	M		PP858704	PP853413	PP887903	PP858726			LTU M43 PAW43
WAMM2811	*S. v. rufigenis*	PNG; Lake Daviumbu	−7.5041	141.1778	1936‐08‐23	M	A							
ANWCM16260	*S. v. virginiae*	QLD; East McIlwraith Ra.	−13.8861	143.5028	1990‐08‐09	F	A							
ANWCM16295	*S. v. virginiae*	QLD; East McIlwraith Ra.	−13.8861	143.5028	1990‐08‐13			PP858698	PP853404	PP887891	PP858707	PP887935	PP898282	
ANWCM29830	*S. v. virginiae*	QLD; Heathlands Stn.	−11.8167	142.4000	1992‐03‐09	F	A		PP853402	PP887904	PP858727	PP887934		LTU F1/Q1
ANWCM29831	*S. v. virginiae*	QLD; Heathlands Stn.	−11.8167	142.4000	1992‐03‐09	F			PP853403	PP887905	PP858728			LTU F2/Q2
ANWCM29835	*S. v. virginiae*	QLD; Heathlands Stn.	−11.8167	142.4000	1992‐03‐09	M		AF088952	AF088975	AF088933	PP858711	JQ599229	JQ687038	LTU M6/Q6
ANWCM29837	*S. v. virginiae*	QLD; Heathlands Stn.	−11.8167	142.4000	1992‐03‐09	M	A		PP853414	PP887906	PP858724			LTU M8/Q8
ANWCM29843	*S. v. virginiae*	QLD; Captive bred			1992‐09‐28	M			PP851404	PP887907	PP858740			LTU M14 Q14
ANWCM29850	*S. v. virginiae*	QLD; Captive bred			1992‐10‐24	M			PP853415	PP887908	PP858732			LTU M21 Q21
ANWCM29851	*S. v. virginiae*	QLD; Captive bred			1992‐10‐24	M				PP887909	PP898286			LTU M22 Q22
ANWCM739	*S. v. virginiae*	QLD; Massy Creek Scrub	−13.9333	143.5000	1964‐07‐17	F	A							
AWCSvirg	*S. v. virginiae*	QLD; Piccaninny Plains	−13.2906	142.7803	2023‐07‐23			PP858700	PP853417	PP887911	PP858709			
QMJ15890	*S. v. virginiae*	QLD; Herbert Vale	−18.4667	145.8500		M	A							
QMJ3109	*S. v. virginiae*	QLD; Hampden	−21.0667	149.0000	1917‐09‐21	M	A							
QMJM1000	*S. v. virginiae*	QLD; Abergowrie	−18.4833	145.8833	1970‐06‐19	F	A							
QMJM10552	*S. v. virginiae*	QLD; Cooktown	−15.4667	145.2500	1993	F	A							
QMJM17095	*S. v. virginiae*	QLD; Cape Flattery	−14.9250	145.2250	1986‐09‐05	M	A							
QMJM17250	*S. v. virginiae*	QLD; Cooktown	−15.4611	145.0922				PP858696	PP853425	PP887919				
QMJM18722	*S. v. virginiae*	QLD; Mt Ray Stn	−15.0736	145.0228	2007‐08‐23			PP858697	PP853426	PP887920	PP858712	PP887940		
QMJM4012	*S. v. virginiae*	QLD; Mapoon Plains	−11.9844	141.8681	1980‐09‐06	M	A							
QMJM4013	*S. v. virginiae*	QLD; Mapoon Plains	−11.9844	141.8681	1980‐09‐06	M	A							
QMJM4015	*S. v. virginiae*	QLD; Mapoon Plains	−11.9844	141.8681	1980‐09‐08	M	A							
QMJM4017	*S. v. virginiae*	QLD; Mapoon Plains	−11.9844	141.8681	1980‐09‐08	M	A							
QMJM4018	*S. v. virginiae*	QLD; Pine R Bay	−12.5417	141.7083	1981‐02‐21	M	A							
QMJM4019	*S. v. virginiae*	QLD; York Downs	−12.7531	142.3100	1981‐05‐18	M	A							
QMJM5279	*S. v. virginiae*	QLD; Shiptons Flat	−15.8000	145.2667	1986‐12‐31	M	A							
QMJM689	*S. v. virginiae*	QLD; Home Rule	−15.7500	145.2833	1974‐11‐07	F	A							
QMJM7333	*S. v. virginiae*	QLD; Mission Beach	−17.9500	146.1000	1977‐06‐04	F	SA							
QMJM998	*S. v. virginiae*	QLD; Helenvale	−15.7000	145.2167	1975‐03‐27	F	A							
Outgroups														
	*S. archeri*	QLD						AF088937	AF088961	AF088920	DQ157410	JQ599241	JQ687050	
	*S. archeri*	PNG							PP900178					NMVZ33147
	*S. crassicaudata*							JN714278	L28087	N 007631	AY014770	JQ599231	EF028748	
	*S. bindi*	NT; Coomalie							AF088962	AF088921			JQ687040	
	*S. bindi*	NT; Pine Creek						AF088938		KF294263				
S. douglasi 1	*S. douglasi*	QLD						AF088941	AF088965	AF088923		JQ599232	JQ687041	
S. douglasi 2	*S. douglasi*	QLD						NC006517	NC006517	NC006517				
	*S. macroura*	QLD						AF088948	AF001935	AF001582		JQ599230	JQ687039	MW 40/89
	*S. macroura*	WA						MT366306	AF339114	MT440157	MT441411	MT454753		

Abbreviations: ABTC, Specimens and tissue samples sourced from the Australian Biological Tissue Collection; ANWC the Australian National Wildlife Collection; AWC, the Australian Wildlife Conservancy; DBCA, the Western Australian Department of Biodiversity, Conservation and Attractions; MAGNT, the Museum and Art Gallery of the Northern Territory; NMV, Museums Victoria; QM, the Queensland Museum; WAM, the Western Australian Museum.

^a^ Dunkeld Pastoral Co.

**TABLE A2:** Models of sequence evolution obtained for the Bayesian analyses of the *Sminthopsis* sequence data.

Gene	Model	γ value	p invar
IRBP	GTR + G	0.1700	–
bFIB7	GTR + I + G	0.2900	–
ω‐globin	GTR + G	5.3730	–
CR	GTR + I+ G	0.4810	0.1430
CYTB	GTR + I + G	1.3750	0.5400
12SrRNA	GTR + I + G	0.0000	–
Concatenated			
Nuclear	GTR + G	0.2390	–
Mitochondrial	GTR + I + G	0.6650	–
Protein coding (Pos1 + 2)	GTR + I + G	0.4300	0.0320
Protein‐coding (3rd Pos RY)	GTR + I + G	0.5400	0.0000
Introns	GTR + I + G	0.5030	0.0110
12S stems	GTR + I + G	0.1080	0.3230
12S loops	TIM2 + I + G	0.9660	0.5030
CR	GTR + I + G	0.4810	0.1430

**TABLE A3:** External measurement data (in mm) for *Sminthopsis virginiae* specimens in this study.

Specimen_ID	Taxon	Sex	HV	TL	PES	EAR	HD
ANWCM24722	*nitela*	F	90	87	17.37	18.17	29.98
ANWCM29853	*nitela*	F	90	91	18.23	21.72	30.95
ANWCM29861	*nitela*	M		120	21.34	22.67	
MAGNTU0357	*nitela*	F	85	89	16.92	19.92	28.89
MAGNTU0529	*nitela*	F	80	87	18.40	19.66	30.01
MAGNTU2034	*nitela*	M	87	107	19.31		29.68
MAGNTU3771	*nitela*	F	90	103	19.11	18.01	30.32
MAGNTU3772	*nitela*	F		99	17.70	20.52	
MAGNTU4129	*nitela*	M	94	110	20.27	21.54	31.80
MAGNTU4190	*nitela*	M		103	18.61	23.03	
MAGNTU4325	*nitela*	M	86	102	18.62	20.20	30.64
MAGNTU4855	*nitela*	F	82	100	18.99	18.31	30.74
MAGNTU5136	*nitela*	M	84	90	19.10	18.90	30.23
MAGNTU5219	*nitela*	F	80	88	17.64		29.05
MAGNTU5353	*nitela*	F	76	98	17.63	20.97	29.05
MAGNTU5877	*nitela*	F	110	98	18.89		33.18
MAGNTU5878	*nitela*	M	110	115	20.98		34.74
WAMM15785	*nitela*	F	79	99	18.36	18.01	
WAMM15786	*nitela*	F	77	93	17.81	16.43	
WAMM21514	*nitela*	F	83	100	17.24	18.19	
WAMM21515	*nitela*	M	73	102	18.99	18.15	
WAMM21601	*nitela*	F	85	95	18.43	17.97	30.65
WAMM21771	*nitela*	F	90	105	18.40	18.33	30.88
WAMM21772	*nitela*	F	75	93	17.66	18.04	27.51
WAMM21789	*nitela*	M	84	103	18.11		29.70
WAMM21861	*nitela*	M	74	90	18.22	17.37	29.91
WAMM21893	*nitela*	M	86	102	18.99	16.82	30.24
WAMM21901	*nitela*	M	91	94	17.93	16.85	30.26
WAMM21910	*nitela*	F	80	86	17.44	18.24	27.72
WAMM21911	*nitela*	F	92	97	17.68	18.46	30.83
WAMM21925	*nitela*	M	83	98	18.56	18.45	29.42
WAMM21926	*nitela*	M	81	96	19.19	18.60	29.44
WAMM21988	*nitela*	F	93	101	18.33	19.75	29.68
WAMM21989	*nitela*	F	91	104	19.39	17.24	31.55
WAMM21996	*nitela*	M	85	94	18.52	16.82	30.87
WAMM22067	*nitela*	F	80	95	18.61	19.23	29.64
WAMM22068	*nitela*	F	80	104	19.52	20.47	29.23
WAMM25796	*nitela*	F	100	95	17.50		29.93
WAMM4056	*nitela*	M	96	84	19.57	19.47	33.32
WAMM49752	*nitela*	M	80	107	19.61	18.91	31.10
WAMM52678	*nitela*	M	85	100	18.91	19.57	29.13
WAMM54888	*nitela*	F	95	103	18.05	19.77	30.58
WAMM54891	*nitela*	F	78	101	15.85	18.01	27.68
WAMM54892	*nitela*	F	88	104	17.73	19.55	30.94
WAMM60906	*nitela*	M	75		17.84	17.83	29.06
WAMM60929	*nitela*	M		109	17.25	17.65	30.72
WAMM60930	*nitela*	M		90	17.47	13.67	27.06
ANWCM29776	*rufigenis*	M	93		21.14	18.5	
ANWCM29777	*rufigenis*	F	110	111	21.8	19.69	33.97
ANWCM29779	*rufigenis*	M	108	104	23.1	20.76	35.26
ANWCM29780	*rufigenis*	F	94	90	20.18	17.6	31.59
ANWCM29782	*rufigenis*	F	104	102	21.76	18.57	35.42
ANWCM29784	*rufigenis*	M	108	116	23.5	22.28	36.21
ANWCM29785	*rufigenis*	M	105	109	21.07	19.36	34.79
ANWCM29786	*rufigenis*	M		112	20.76	19.01	
ANWCM29789	*rufigenis*	M	110	118	23.14	22.94	37.86
ANWCM29791	*rufigenis*	F	102	112	20.87	21.23	34.52
ANWCM16260	*virginiae*	F	110	106	20.45	17.53	32.72
ANWCM29830	*virginiae*	F	103	97	20.99	20.95	34.41
QMJ15890	*virginiae*	M		98	20.6	18.68	
QMJM1000	*virginiae*	F	97	102.5	20.32	19	34.03
QMJM10552	*virginiae*	F	104.5	113	20.25	19.14	32.05
QMJM17095	*virginiae*	M	103	110	20.77	22.23	33.52
QMJM4012	*virginiae*	M	97.5		22.37	18.13	33.77
QMJM4013	*virginiae*	M	103	105	21.16	20.09	34.37
QMJM5279	*virginiae*	M	113	102	21.94	20.17	33.4
QMJM689	*virginiae*	F	115	111	20.05	19.19	
QMJM7333	*virginiae*	F	93	103	20.38	18.28	30.88
QMJM998	*virginiae*	F		119	21.26	19.83	33.16

Abbreviations: EAR, ear length; HD, head length; HV, Head‐vent length; PES, hind‐foot length; TL, tail‐vent length.

**TABLE A4:** ANOVA post hoc test scores for each of the cranial measurements, except for ATL for which a Kruskal–Wallis test was run instead.

Comparison pair	BL	FL	NL	PML	NWA	NWP	RWP	IOW	UIL
*rufigenis‐nitela*	**	***	ns	ns	*	*	***	**	***
*virginiae‐nitela*	ns	ns	ns	ns	ns	***	ns	ns	**
*virginiae‐rufigenis*	ns	*	ns	ns	ns	ns	ns	ns	ns

	RWA	IBW	ATL	ATW	OBW	MW	CW	UCML	UML
*rufigenis‐nitela*	***	***	**	ns	ns	**	*	**	***
*virginiae‐nitela*	**	**	ns	ns	ns	*	*	**	***
*virginiae‐rufigenis*	ns	ns	ns	ns	ns	ns	ns	ns	ns

	UPL	M2W	M3W	LIML	LML	LPL	DH	ZW	DL
*rufigenis‐nitela*	**	***	***	***	***	***	ns	*	**
*virginiae‐nitela*	***	***	**	***	***	*	*	*	*
*virginiae‐rufigenis*	ns	ns	ns	ns	ns	ns	ns	ns	ns

*Note*: Significance value codes as ns = *p*‐value >.05; * = *p* < .05; ** = *p* < .01; *** = *p* < .001. Color intensity reflects the strength of the *p*‐value significance, with darker colors having smaler *p*‐values.

**TABLE A5:** Results of k‐nearest neighbor discriminant analysis, showing how many samples were classified into the correct and incorrect groups.

Raw data by subspecies					
Subspecies	*n*	As *nitela*	As *rufigenis*	As *virginiae*	% correct
*nitela*	16	15	1	0	93.8
*rufigenis*	7	0	6	1	85.7
*virginiae*	7	1	3	3	42.9
Total	30	18	7	5	80.0

Size‐corrected data by subspecies					
*nitela*	16	14	0	2	87.5
*rufigenis*	7	1	4	4	57.1
*virginiae*	7	2	0	5	71.4
Total	30	17	4	9	76.7

Raw data by genetic clade				
Subspecies			As *rufigenis* + *virginiae*	
*nitela*	16	15	1	93.8
*rufigenis* + *virginiae*	14	1	13	92.9
Total	30	16	14	93.3

Size‐corrected data by genetic clade				
*nitela*	16	14	2	87.5
*rufigenis* + *virginiae*	14	3	11	78.6
Total	30	17	13	83.3
