# Supplementary material for: Vicariant speciation resulting from biogeographic barriers in the Australian tropics: The case of the red‐cheeked dunnart (Sminthopsis virginiae)
Source: Ecol Evol. 2024 Aug 27;14(8):e70215. doi: 10.1002/ece3.70215 (PMC11349609; doi:10.1002/ece3.70215)
Supplement: Supplementary file 2 — File S2. [file ECE3-14-e70215-s001.docx]

**Supplementary Methods for Umbrello et al. 2024 Vicariant speciation resulting from biogeographic barriers in the Australian tropics: the case of the red-cheeked dunnart (*Sminthopsis virginiae*).**

*Molecular laboratory methods*

Total genomic DNA was obtained from preserved liver, muscle, ear and toe tissue at the Western Australian Museum Molecular Laboratory. Approximately 15 mg of tissue was used to extract DNA in Qiagen DNeasy tissue and blood tube kits according to manufactures’ instructions, and DNA was eluted into 120 µL low-EDTA tris buffer. All regions were amplified via polymerase chain reaction (PCR) in 25 µL reaction volumes containing 1 µL of template DNA, 1x PCR buffer containing 1.5 mM MgCl2 (Applied Biosystems, Branchburg, NJ, USA), 0.3 µM of each primer (Integrated DNA Technologies) and 1 unit of MyHSTAQ DNA polymerase (Applied Biosystems). Primers used for CR, cytb, 12S, ω-globin, bfib7 and IRBP are listed in Table S1.

For all loci except 12S, PCR reactions were performed at the following conditions; 95°C for 5 min, then 35 cycles of denaturation of 95°C for 30 s, annealing at 50°C for 30 to 45 s, extension at 72°C for 60 to 90 s, and followed by a final extension at 72°C for 10 min, as detailed in Umbrello *et al.* (2017). For 12S, PCR cycling conditions follow Krajewski *et al.* (1997) and are as follows: 95 °C for 5 min, then nine low-stringency cycles of 95 °C for 45 s, annealing at 49 °C for 50 s, and extension at 72 °C for 55 s, followed by 25 high-stringency cycles of 95 °C for 45 s, 55 °C for 50 s and extension at 72 °C for 1 min followed by a final extension at 72 °C for 5 min. PCR products were visualised on the E-Gel® Electrophoresis system (Life Technologies, Melbourne, Australia) on pre-cast 2% agarose gels with ethidium bromide.

DNA purification and bi-directional Sanger sequencing was carried out at the Australian Genome Research Facility, Perth, WA. Assembly, editing and alignment of sequences occurred in Geneious Prime (Kearse *et al.* 2012), and only sequence reads of higher than 70% quality were used. Ambiguous base pairs and missing data in the forward-reverse assemblies were checked and scored accordingly and retained as heterozygous sites (including indels) in the nuclear assemblies where appropriate. Primer regions were trimmed from the consensus sequences before alignments were created using the MAFFT plug-in in Geneious (Katoh et al., 2002). The presence of stop codons in coding regions were checked prior to analysis and uploading to Genbank and none were present.

**Table S1:** Primers used for PCR amplification and sequencing

| Name [ref] | Direction | Sequence | Reference |
| --- | --- | --- | --- |
|  |  | Control region (left domain) |  |
| L15999M | FWD | 5'-ACCATCAACACCCAAAGCTGA-3' | Fumagalli *et al.* (1997) |
| H16498M | REV | 5'-CCTGAAGTAGCAACCAGTAG-3' | Fumagalli *et al.* (1997) |
|  |  | Cytochrome b |  |
| MVZ-05 | FWD | 5'-CGAAGCTTGATATGAAAAACCATCGTTG-3’ | da Silva & Patton (1993) |
| L15311 | FWD | 5'-CTACCATGAGGACAAATATC-3' | Helm-Bychowski and Cracraft (1993) |
| MVZ-16 | REV | 5'-AAATAGGAARTATCAYTCTGGTTTRAT-3’ | da Silva & Patton (1993) |
| H15149 | REV | 5'-AAACTGCAGCCCCTCAGAATGATATTTGTCCTCA-3' | Kocher *et al*. (1989) |
| H15767 | REV | 5'-ATGAAGGGATGTTCTACTGGTTG-3' | Edwards *et al.* (1991) |
| H15915A | REV | 5'-AACCTTCGTTGTTGGCTTACAAGAC-3' | Krajewski *et al.* (1997) |
|  |  | 12S rRNA |  |
| L12C | FWD | 5'-AAAGCAAAACACTGAAAATG-3' | Springer *et al*. (1995) |
| H12GG | REV | 5'-TRGGTGTARGCTRRRTGCTTT-3' | Springer *et al*. (1995) |
|  |  | ω-globin intron 2 |  |
| G314 | FWD | 5'-GGAATCATGGCAAGAAGGTG-3' | Wheeler *et al.* (2001) |
| G424 | REV | 5'-CGGAGGTGTTYAGTGGTATTTTC-3' | Wheeler *et al.* (2001) |
|  |  | Bfib7 |  |
| U | FWD | 5'-ATGTACTTCAGCACCTATGA-3' | Krajewski *et al*. (2004) |
| IL | REV | 5'-AATYTTAGTGTTGAGCA-3' | Krajewski *et al*. (2004) |
| L2 | REV | 5'-ACCATCTTCCTTAGAACACTG-3' | Krajewski *et al*. (2004) |
|  |  | IRBP |  |
| G  JJ | FWD | 5'-CTAGTGATCTCTTATGAGCC-3' | Krajewski *et al*. (2004) |
| J | REV | 5'-GCTGCCCTCCMAAGTCTG-3' | Krajewski *et al*. (2004) |

**References**

da Silva MNF, Patton JL (1993). Amazonian phylogeography: mtDNA sequence variation in arboreal echimyid rodents (Caviomorpha). *Molecular Phylogenetics and Evolution* **2**, 243–255.

Edwards SV, Arctander P, Wilson AC (1991). Mitochondrial resolution of a deep branch in the genealogical tree for perching birds. *Proceedings of the Royal Society of London* *B* **243**, 99–107.

Fumagalli L, Pope LC, Taberlet P, Moritz C (1997). Versatile primers for the amplification of the mitochondrial DNA control region in marsupials. *Molecular Ecology* **6**, 1199–1201.

Helm-Bychowski K, Cracraft J (1993). Recovering phylogenetic signal from DNA sequences: relationships within the corvine assemblage (Class Aves) as inferred from complete sequences of the mitochondrial DNA cytochrome-b gene. *Molecular Biology and Evolution* **10**, 1196–1214.

Katoh K, Misawa K, Kuma K, Miyata T (2002). MAFFT: a novel method for rapid multiple sequence alignment based on fast Fourier transform. *Nucleic Acids Research* **30**, 3059–3066.

Kearse M, Moir R, Wilson A, Stones-Havas S, Cheung M, Sturrock S, Buxton S, Cooper A, Markowitz S, Duran C, Thierer T, Ashton B, Meintjes P, Drummond A (2012). Geneious Basic: an integrated and extendable desktop software platform for the organization and analysis of sequence data. *Bioinformatics (Oxford, England)* **28**, 1647–9. doi:10.1093/bioinformatics/bts199

Krajewski C, Blacket M, Buckley L, Westerman M (1997). A multigene assessment of phylogenetic relationships within the dasyurid marsupial subfamily Sminthopsinae. *Molecular Phylogenetics and Evolution* **8**, 236–248. doi:10.1006/mpev.1997.0421

Krajewski C, Moyer GR, Sipiorski JT, Fain MG, Westerman M (2004). Molecular systematics of the enigmatic ‘phascolosoricine’ marsupials of New Guinea. *Australian Journal of Zoology* **52**, 389–415.

Kocher TD, Thomas WK, Meyer A, Edwards SV, Pääbo S, Villablanca FX, Wilson AC (1989). Dynamics of mitochondrial DNA evolution in animals: Amplification and sequencing with conserved primers. *Proceedings of the Natural Academy of Science USA* **86**, 6196–6200.

Springer MS, Hollar LJ Burke A (1995). Compensatory substitutions and the evolution of the mitochondrial 12S rRNA gene in mammals. *Molecular Biology and Evolution* **12**, 1138–1150.

Umbrello LS, Woolley PA, Westerman M (2017). Species relationships in the dasyurid marsupial genus *Pseudantechinus* (Marsupialia: Dasyuridae): a re-examination of the taxonomic status of *Pseudantechinus roryi*. *Australian Journal of Zoology* **65**, 240–247. doi:10.1071/ZO17059
